# Effects of Curing Schedules on Carbon-Fiber-Reinforced Laminates with a Bio-Based Epoxy Matrix

**DOI:** 10.3390/polym18172154

**Published:** 2026-09-03

**Authors:** Larisa-Anda Stroe, Daniel-Eugeniu Crunteanu, Mihail Botan, Adriana Stefan, George Catalin Cristea, Gabriela-Liliana Stroe

**Affiliations:** 1 Department of Aerospace Sciences, National University of Science and Technology POLITEHNICA Bucharest, Splaiul Independenţei 313, 060042 Bucharest, Romania; larisa_anda.stroe@upb.ro (L.-A.S.);; 2 INCAS—National Institute for Aerospace Research “Elie Carafoli”, B-dul Iuliu Maniu 220, 061126 Bucharest, Romania

**Keywords:** bio-based epoxy resin, carbon-fiber-reinforced polymers (CFRPs), sustainable composites, curing schedule, epoxy matrix composites

## Abstract

Carbon-fiber-reinforced polymer (CFRP) composites fabricated with bio-based epoxy resins represent a promising approach for sustainable lightweight structures produced by out-of-autoclave (OoA) technologies. The curing schedule influences the state of the epoxy matrix and, consequently, can affect the fiber–matrix interaction and laminate performance. This study investigates the effect of practical curing conditions on 2 × 2 twill woven carbon fiber laminates fabricated by vacuum infusion using a commercially available bio-based epoxy resin IB2. The manufacturer’s recommended room-temperature conditions (25 °C for 24 h) were compared with accelerated mold heating schedules at 40, 50, 60, and 70 °C for 12 h. The laminates were characterized by three-point tensile and flexural tests, heat deflection temperature (HDT) measurements, differential scanning calorimetry (DSC), and SEM fractography. The tensile response showed limited sensitivity to the investigated curing conditions, with mean tensile strengths ranging from 634.01 to 672.95 MPa; T60 exhibited the highest mean numerical tensile strength (672.95 ± 53.60 MPa) and tensile modulus (53.39 ± 11.53 GPa), although the differences were small relative to the experimental spread. In contrast, the flexural response was more sensitive to the processing conditions. T70 exhibited the highest average flexural strength (980.60 ± 129.03 MPa), strain at maximum flexural stress, and strain energy density to maximum stress (8.75 ± 1.89 MJ/m^3^). The heat deflection temperature (HDT) systematically increased from 65.20 °C for T25 to 85.83 °C for T70. DSC revealed clear differences in the calorimetric response after curing during the first heating cycle, while the glass transition temperatures at the middle of the second heating occupied a relatively narrow range of 80.9–85.6 °C. SEM fractography revealed mixed tensile failure mechanisms related to fibers, matrix, and interface under all curing conditions. Overall, the results demonstrate that accelerated 12 h heated mold programs can reduce cure time while maintaining tensile performance generally comparable to the 24 h room-temperature IB2 reference condition and providing higher average flexural performance and thermal deformation resistance under load. These findings establish processing–property relationships relevant to the development of biomass-based CFRP OoA laminates for lightweight aerospace applications.

## 1. Introduction

According to the Flightpath 2050 and Clean Aviation strategic frameworks, future aerospace structures must simultaneously achieve significant reductions in weight, energy consumption, greenhouse gas emissions, and environmental footprint, while maintaining the high levels of safety and reliability required by aeronautical applications [1,2,3,4]. These requirements are necessary for both aircraft and unmanned aerial vehicles (UAVs), where structural mass directly affects energy consumption and mission capability [3,4,5]. At the same time, environmental pollution associated with petroleum-derived thermoset matrices has stimulated the development of partially or fully biobased epoxy systems as potential alternatives for structural composites [6,7,8,9,10,11,12,13]. However, their practical implementation requires not only suitable intrinsic properties, but also processing conditions capable of providing reliable mechanical and thermal performances.

Carbon-fiber-reinforced polymers (CFRPs) are currently among the most widely used structural materials in aerospace engineering because of their exceptional specific stiffness, specific strength, fatigue resistance, corrosion resistance, and design flexibility [14,15]. The replacement of conventional metallic structures with fiber-reinforced composites can provide substantial mass savings. Reductions of approximately 15–45% compared with aluminum have been reported for UAV structures, while CFRP weight savings of about 20% for primary aerospace structures and up to 40% for secondary structures have been reported [15,16]. Such mass reduction directly benefits UAV performance: a 10% decrease in UAV weight has been estimated to improve operational efficiency by approximately 6–8%, with corresponding reductions in fuel or electrical power consumption [5]. The resulting mass margin can consequently be exploited to increase payload capacity or flight endurance, which is particularly important for UAV applications [5,15]. Consequently, CFRP materials are increasingly employed in fuselage structures, wings, propellers, payload supports, battery enclosures, and multifunctional aerospace components [17,18,19,20].

The mechanical response of polymer composites is also strongly dependent on the reinforcement architecture. Short fibers offer greater processing flexibility, but their load transfer capacity is strongly influenced by fiber length and orientation, while continuous fibers offer more efficient load transfer and are therefore preferred for high-performance structural laminates. Recent studies further demonstrate that curing conditions are an established but highly system-dependent processing variable. Liang et al. showed that curing temperature significantly affects the mechanical performance and fiber–matrix interaction of conventional carbon/epoxy composites [21], while Ferdosian et al. correlated the curing kinetics of lignin-based epoxy systems with their resulting mechanical properties [22]. More recently, Sam-Daliri et al. demonstrated that the curing/fabrication process also significantly influences the mechanical response of continuous fiber epoxy composites [23]. These findings indicate that the general effect of curing is well established, but its quantitative influence remains dependent on the specific matrix chemistry, reinforcement architecture, and manufacturing process.

In this context, the study aims to establish a controlled relationship between process, structure and properties for CFRP laminates made with a commercially available bio-based epoxy resin and reinforced with continuous carbon fibers. Vacuum-infused laminates, with the same reinforcement architecture and comparable fiber volume fractions, were cured at temperatures ranging from 25 to 70 °C and evaluated by tensile and flexural tests, differential scanning calorimetry (DSC), heat deflection temperature (HDT) and Scanning Electron Microscopy (SEM) fractography. A room-temperature cure recommended by the manufacturer was used as a reference and compared with accelerated heating programs designed to reduce the processing time from 24 h to 12 h. Through this combined approach, differences in the calorimetric response associated with the investigated curing schedules are considered together with fracture-surface morphology and macroscopic mechanical response. Particular attention is paid to evaluating whether a single curing schedule can provide favorable tensile and flexural behavior together with increased resistance to thermal deformation under load, thus providing a basis for selecting practical curing schedules according to the structural requirements pursued.

## 2. Materials and Methods

### 2.1. Materials and Composite Manufacturing

**Figure 1:**
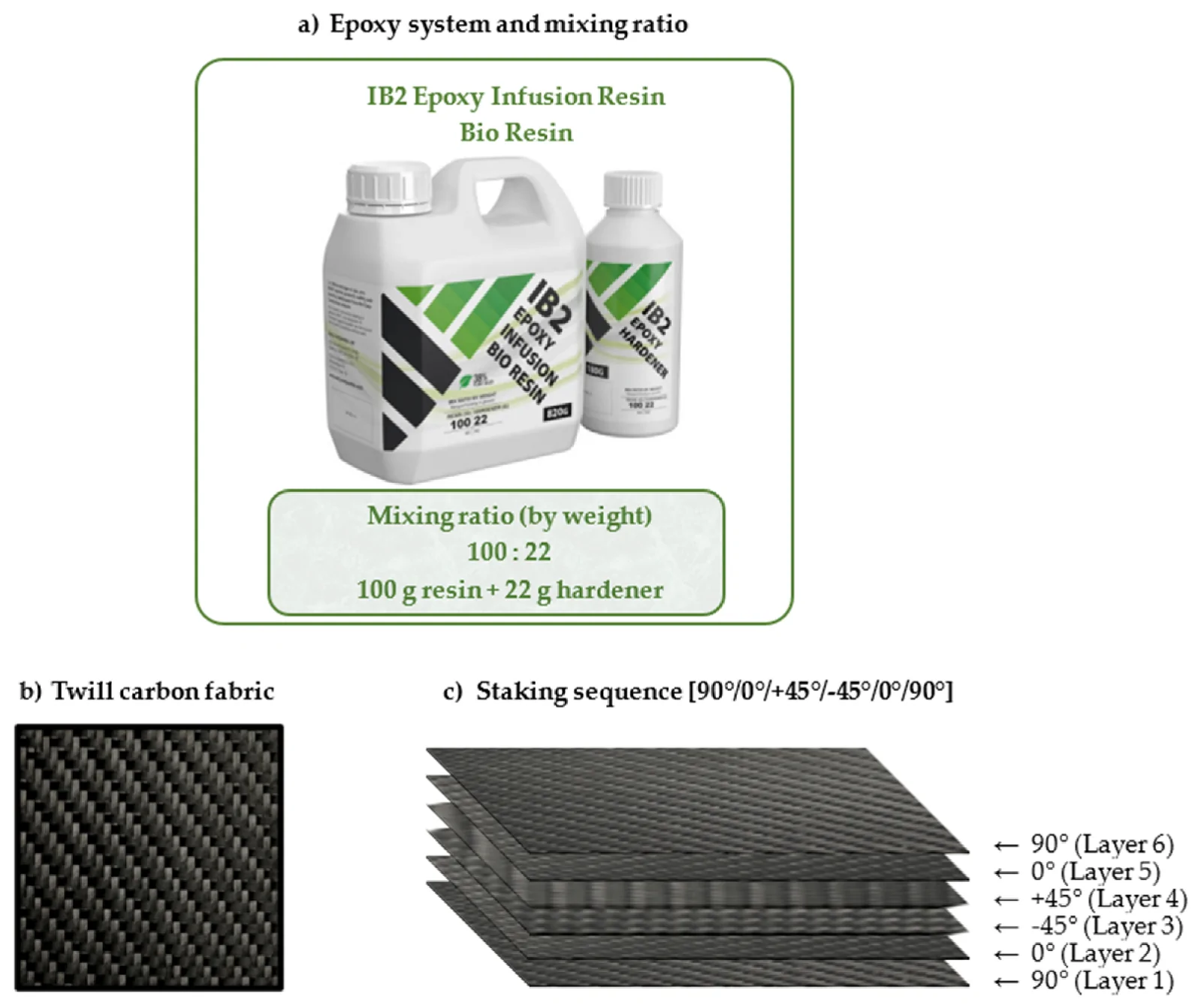
Materials used in the study and laminate architecture: (**a**) epoxy system and mixing ratio; (**b**) carbon fiber twill fabric; (**c**) stacking sequence.

The bio-resin-based epoxy CFRP laminates were fabricated as flat panels by vacuum infusion, from which specimens were subsequently cut to evaluate the influence of curing temperature on their mechanical and thermal behavior. The matrix system consisted of IB2 Infusion Bio Resin epoxy resin supplied by Easy Composites Ltd. (Stoke-on-Trent, UK), used together with the appropriate hardener. According to the manufacturer’s recommendations, the resin and hardener were mixed in a weight ratio of 100:22, corresponding to 100 g of resin and 22 g of hardener (Figure 1a). The selected matrix system is a two-component epoxy resin, specifically developed for vacuum infusion applications. According to the manufacturer’s data sheet, the resin contains approximately 38% plant-derived carbon content. The mixed resin system has a viscosity of approximately 290 mPa·s at 20 °C and a density of 1.12 g/cm^3^. The mixed resin system has a low viscosity of approximately 290 mPa·s at 20 °C and a density of 1.12 g/cm^3^, providing excellent impregnation and wetting characteristics for the fiber reinforcements during the infusion process.

The reinforcement consisted of a 2 × 2 twill-woven carbon fiber fabric (Figure 1b), supplied by Easy Composites Ltd. (Stoke-on-Trent, UK), with an areal weight of 200 g/m^2^ per ply. The carbon fibers were selected due to their high stiffness and specific strength, making them suitable for lightweight structural composite applications. The density of the carbon fibers was 1.80 g/cm^3^, according to the manufacturer’s reported data. The main physical characteristics of the raw materials used in this study are summarized in Table 1. The values were taken from the manufacturers’ data sheets and were used as reference parameters during the fabrication of the composites.

The composite panels were manufactured from six plies of woven carbon-fiber fabric, manually arranged according to the multidirectional stacking sequence [90°/0°/+45°/−45°/0°/90°], as illustrated in Figure 1c. This configuration was selected in order to provide a balanced in-plane mechanical response while reducing orientation-dependent effects during loading.

Following completion of the lay-up, the vacuum-bagging assembly was prepared using peel ply, flow media, and standard infusion consumables. Resin infusion was performed at a vacuum level of approximately −0.7 bar until complete impregnation of the fiber preform was achieved. After impregnation, the laminates were subjected to controlled curing conditions. All laminates were manufactured using the same reinforcement architecture and identical vacuum-infusion processing parameters, resulting in comparable fiber volume fractions. The processing variables were curing temperature and curing time, which were not varied independently in the present experimental design. Accordingly, the investigated conditions are treated as distinct curing schedules rather than as an independent assessment of curing-temperature effects.

**Figure 2:**
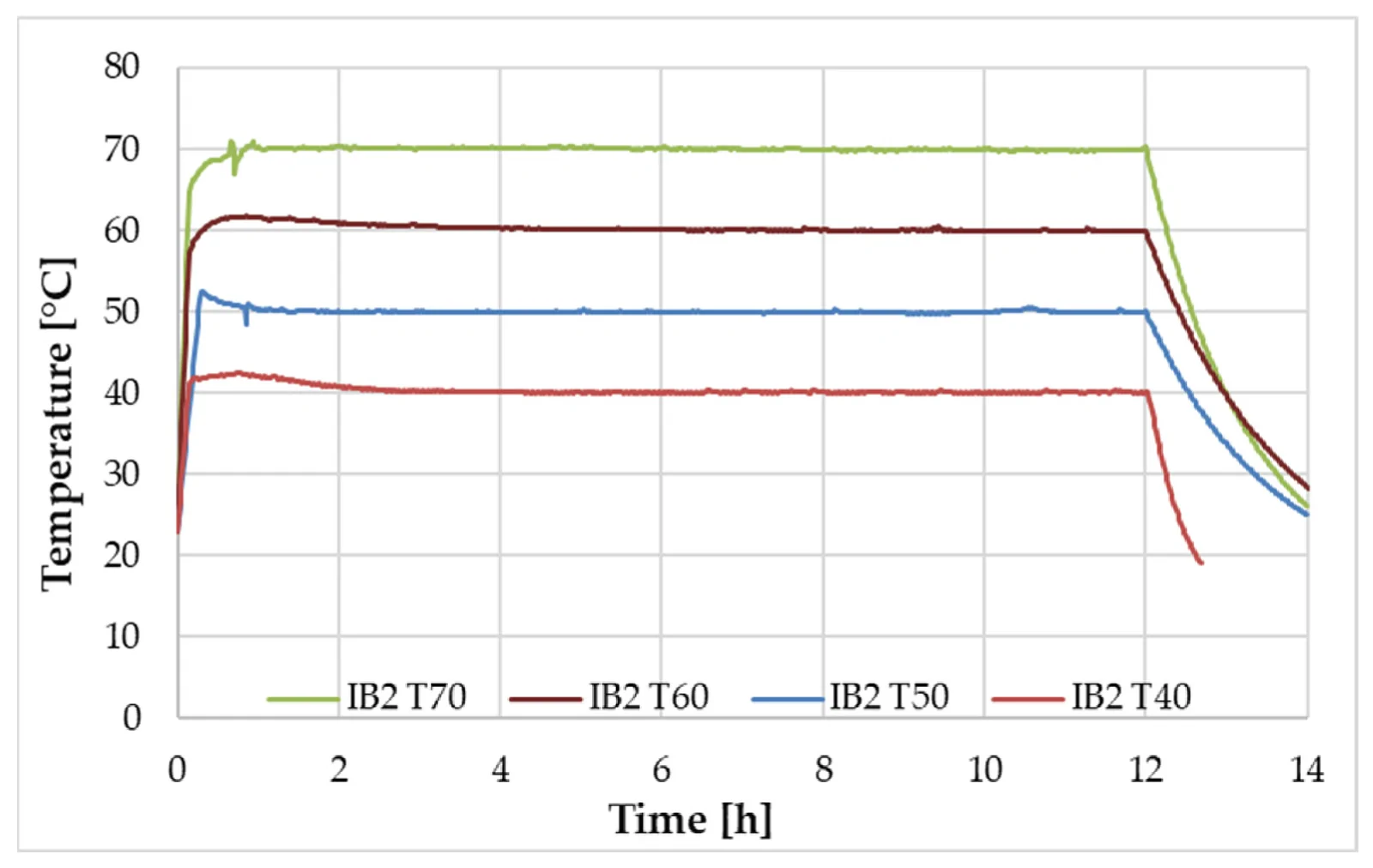
Temperature profiles recorded by thermocouples placed on the mould surface during the 12 h heated-mould curing cycles at 40, 50, 60, and 70 °C.

Five curing conditions, designated T25, T40, T50, T60, and T70, were investigated, corresponding to curing temperatures of 25 ± 2 °C, 40 ± 2 °C, 50 ± 2 °C, 60 ± 2 °C, and 70 ± 2 °C, respectively. The T25 laminates were cured for 24 h at room temperature, whereas the T40–T70 laminates were cured for 12 h on temperature-controlled heated molds. Thermocouples placed on the mold surfaces were used to continuously monitor the curing temperature, while thermal insulation blankets were employed to reduce heat losses and improve temperature uniformity. The temperature profiles recorded during the 12 h curing cycles at 40, 50, 60, and 70 °C are presented in Figure 2.

For all heated-mold conditions, the recorded temperature increased rapidly during the initial heating stage and subsequently remained close to the corresponding target temperature throughout the curing period, confirming stable thermal control of the mould during processing. After completion of the 12 h curing period, the heating system was switched off and the molds were allowed to cool naturally, as indicated by the decreasing temperature profiles at the end of the curves. No thermocouple profile is reported for T25 because this condition corresponded to the manufacturer-recommended 24 h room-temperature cure and was performed under ambient laboratory conditions without active mould heating. After curing, all laminates were cooled to room temperature and conditioned under laboratory conditions for seven days before specimen preparation and testing.

**Figure 3:**
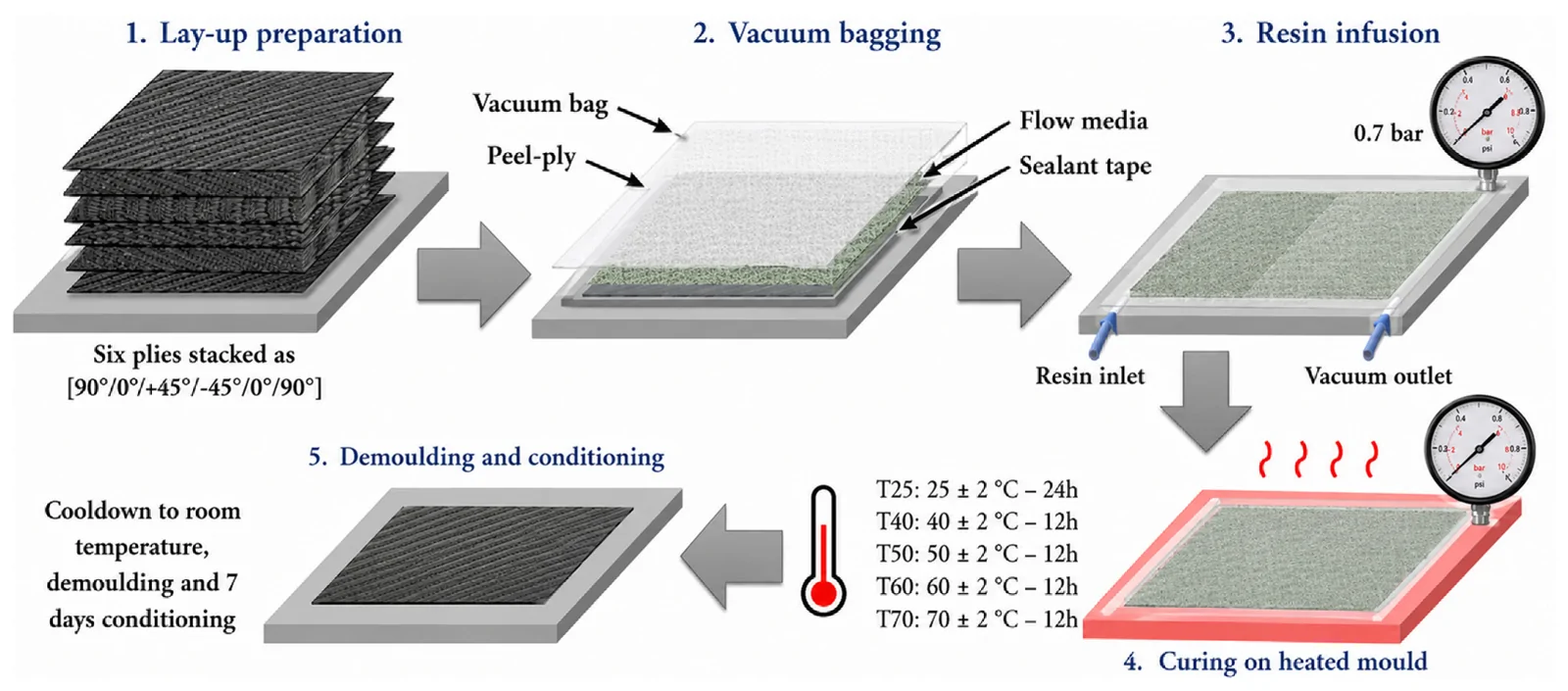
Schematic representation of the resin infusion manufacturing process and curing schedules.

The overall manufacturing procedure is illustrated in Figure 3.

The theoretical fiber volume fraction was estimated from the average dimensions and mass of the specimens prepared for HDT testing for each curing condition. The calculation was based on specimen geometry and mass, with the fiber mass determined from the known areal weight of the fabric, the number of plies, and the specimen area. The resin mass was calculated as the difference between the measured specimen mass and the estimated fiber mass. Assuming negligible void content and a uniform resin distribution throughout the laminate, the fiber volume fraction was calculated using Equations (1)–(3).

The fiber mass corresponding to each specimen was estimated from the projected specimen area using Equation (1):

(1) $$m_{f}=n\cdot A_{d}\cdot A$$

where $m_{f}$ is the estimated fiber mass, n=6 is the number of carbon fiber plies, $A_{d}=200\;g/m^{2}$ is the areal weight of one carbon fabric ply and A is the projected specimen area.

The resin mass retained in the laminate was calculated using Equation (2):

(2) $$m_{r}=m_{s}-m_{f}$$

where $m_{r}$ is the estimated resin mass and $m_{s}$ is the measured mass of the cured composite specimen.

The theoretical fiber volume fraction was calculated using Equation (3):

(3) $$V_{f}=\frac{m_{f}/\rho_{f}}{\left(m_{f}/\rho_{f})+(m_{r}/\rho_{r}\right)}$$

where $V_{f}$ is the theoretical fiber volume fraction $\rho_{f}=1.80\;g/cm^{3}$ is the density of the carbon fibers and $\rho_{r}=1.12\;g/cm^{3}$ is the density of the IB2 epoxy resin system at 20 °C, according to the manufacturers’ datasheets. The calculated fiber volume fractions for the five curing conditions are presented in Table 2.

### 2.2. Specimen Preparation and Experimental Equipment

After curing, the composite laminates were cut into standardized specimens using a Wazer Pro abrasive waterjet cutting system (Wazer, Inc., Brooklyn, NY, USA), minimizing thermal effects on the epoxy matrix and preserving the integrity of the fiber–matrix interface. Prior to testing, all specimens were visually inspected and their dimensions were verified using digital measuring instruments. The experimental program included quasi-static mechanical testing, differential scanning calorimetry (DSC), heat deflection temperature (HDT) evaluation, and morphological characterization. Tensile and flexural tests were performed using an Instron 5982 universal testing machine (Instron, Norwood, MA, USA) equipped with Bluehill^®^ software, Universal 4.08 versionfor data acquisition and analysis. Tensile strain was measured using a non-contact video extensometer. Heat deflection temperature (HDT) measurements were performed using a Qualitest HDT/Vicat testing system (Qualitest International Inc., Plantation, FL, USA). Thermal characterization was carried out by differential scanning calorimetry (DSC) using a NETZSCH DSC 300 Caliris^®^ Classic calorimeter (NETZSCH-Gerätebau GmbH, Selb, Germany), Proteus Thermal Analysis 9.5.3 version, while morphological characterization was performed using a Quanta 250 scanning electron microscope (FEI, Hillsboro, OR, USA), xT 6.2.11.3381 version. The characterization techniques, experimental equipment, and corresponding testing standards are summarized in Table 3.

### 2.3. Mechanical Characterization

#### 2.3.1. Tensile Testing

**Figure 4:**
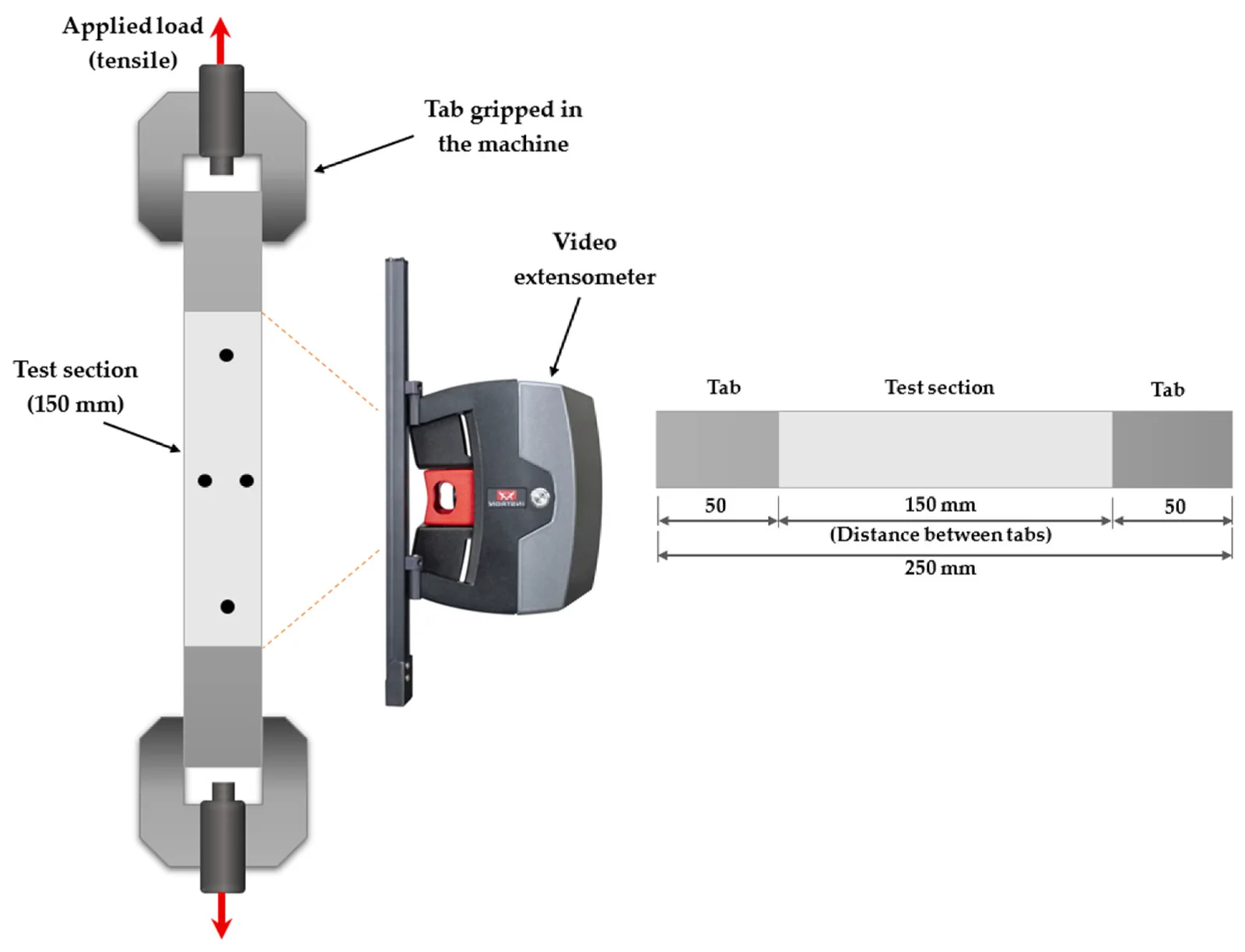
Geometry of the tensile specimens and testing configuration employed for tensile characterization according to ISO 527-4. The red arrows indicate the direction of the tensile load applied at both ends of the specimen.

The tensile behavior of the composite laminates was evaluated according to ISO 527-4 using an Instron 5982 universal testing machine equipped with a non-contact video extensometer. The specimens had a nominal overall length of 250 mm, a width of 25 mm, and a gauge length of 150 mm. The specimen thickness corresponded to the as-manufactured laminate thickness and was approximately 1.23 ± 0.02 mm. All tests were performed under displacement control at a crosshead speed of 2 mm·min^−1^. For each curing condition, at least five specimens were tested, and the results are reported as mean values together with the corresponding standard deviations. Rectangular specimens with bonded tabs at both ends were used to ensure proper load transfer and to reduce the risk of premature failure within the gripping regions. The distance between the inner edges of the tabs was 150 mm, defining the gauge section used for strain measurement with the video extensometer. A schematic representation of the specimen geometry, tab arrangement, and tensile-test configuration is provided in Figure 4.

#### 2.3.2. Flexural Testing

The flexural properties were evaluated using a three-point bending configuration based on ISO 14125. Rectangular specimens with nominal dimensions of 125 mm × 15 mm and an as-manufactured laminate thickness of approximately 1.23 mm were tested using a support span of 96 mm. All tests were performed under displacement control at a crosshead speed of 2 mm·min^−1^.

**Figure 5:**
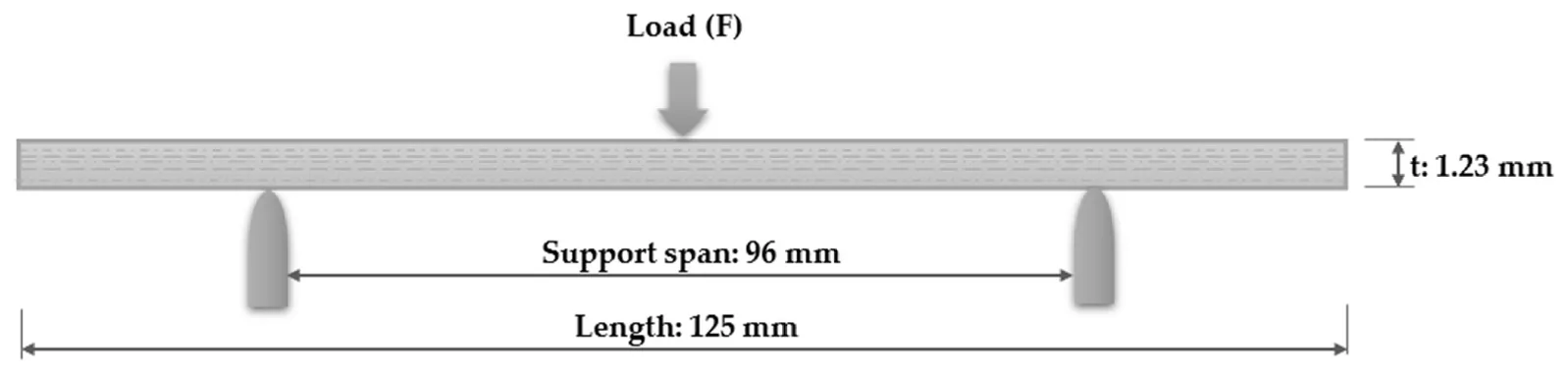
Three-point bending configuration and specimen geometry employed for flexural characterization.

The geometry of the flexural specimens and the testing configuration adopted for three-point bending measurements are illustrated in Figure 5. The schematic provides a visual representation of the specimen dimensions, loading conditions and support span used throughout the experimental programme.

### 2.4. Heat Deflection Temperature (HDT) Evaluation

The resistance of the composite laminates to thermal deformation under load was evaluated by heat deflection temperature (HDT) measurements using a procedure adapted from ASTM D648. Rectangular specimens with nominal dimensions of 100 ± 0.2 mm in length, 32 ± 0.2 mm in width, and 1.23 ± 0.2 mm in thickness were tested using a Qualitest HDT/Vicat system under a nominal flexural stress of 0.45 MPa. During testing, the temperature was increased at a controlled heating rate of 2 °C·min^−1^, while specimen deflection was continuously monitored.

**Figure 6:**
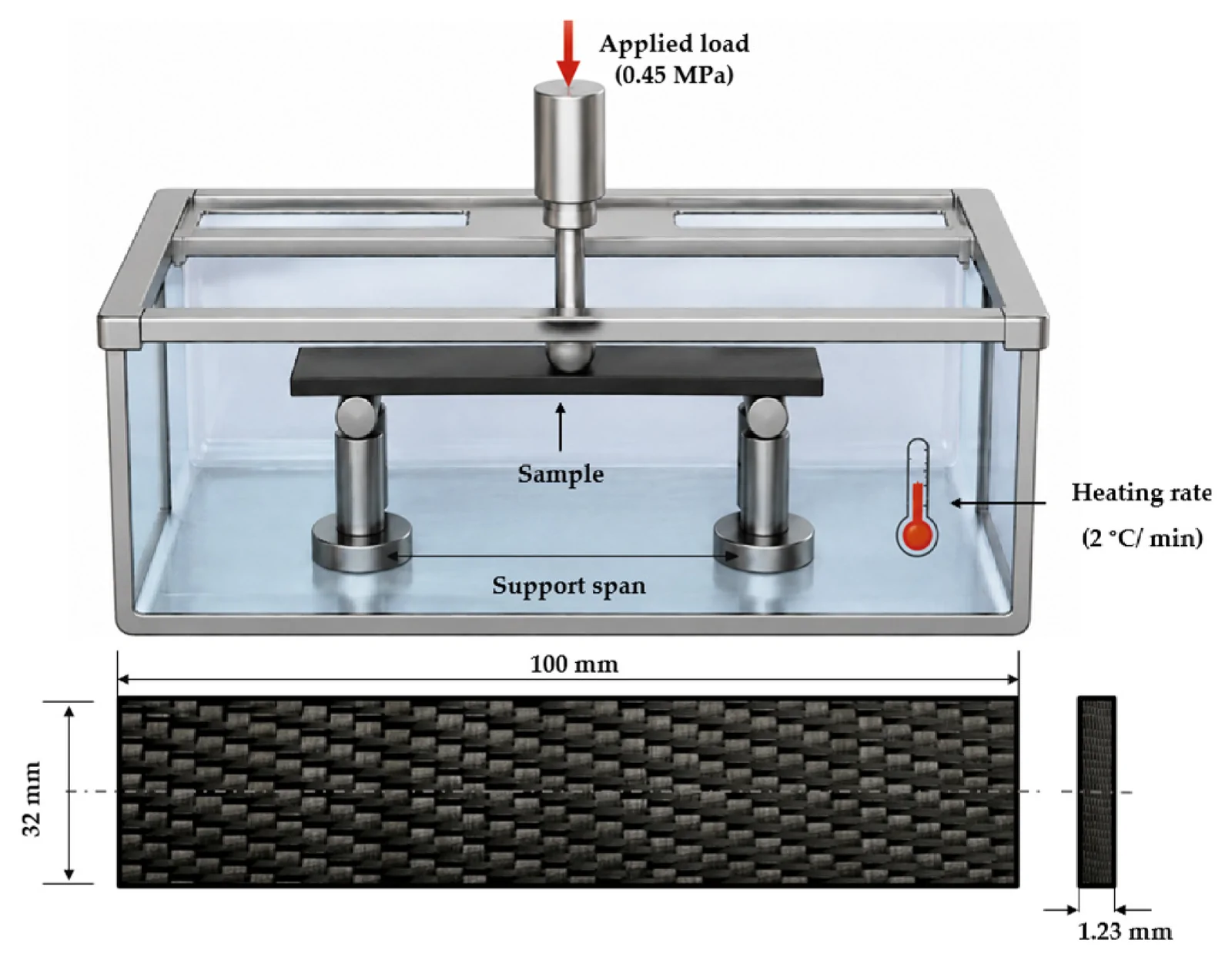
HDT test configuration and specimen geometry used to evaluate thermal deformation under load. The dashed line indicates the longitudinal centerline of the specimen.

The HDT measurements were used to evaluate the influence of the curing conditions on the resistance of the laminates to thermal deformation under load. For comparison among the curing conditions, the HDT value was defined as the temperature at which the specimen reached a deflection of 0.10 mm. A schematic representation of the experimental HDT configuration is presented in Figure 6, including the specimen geometry, loading arrangement, and thermal conditions applied during testing.

### 2.5. Thermal Characterization (DSC)

The thermal behavior of the manufactured CFRP laminates was investigated by differential scanning calorimetry (DSC) using a NETZSCH DSC 300 Caliris^®^ Classic calorimeter equipped with a DSC300 Maia furnace. Composite specimens with masses of approximately 8–9 mg were placed in aluminum Concavus crucibles with pierced lids and analyzed under an inert argon atmosphere. The DSC measurements consisted of two consecutive heating cycles from 30 °C to 200 °C at a heating rate of 10 °C·min^−1^. The first heating cycle was used to characterize the thermal response of the laminates in their as-cured state and to assess differences associated with the initial curing and processing history. This first thermal cycle also removed most of the previous thermal-history effects prior to the second heating. The glass transition temperature (Tg) was subsequently determined from the midpoint of the heat capacity step during the second heating cycle.

### 2.6. SEM Fractographic Analysis

The fracture morphology of the CFRP laminates was investigated by scanning electron microscopy (SEM) using a Quanta 250 microscope (FEI, Hillsboro, OR, USA). Representative fragments were collected from the tensile-fracture regions of specimens corresponding to all five curing conditions (T25, T40, T50, T60, and T70). The fractured surfaces were examined without mechanical polishing in order to preserve the damage features generated during tensile failure.

SEM observations were performed under high-vacuum conditions using secondary-electron imaging. Micrographs acquired at nominal magnifications of 200× and 800× were used to examine the overall fracture morphology and local failure features, respectively. Additional micrographs were acquired at 5000× for local characterization of exposed carbon fibers and for semi-quantitative analysis of residual resin coverage. Particular attention was given to observable features including fiber fracture, fiber pull-out, fiber–matrix separation, matrix cracking, interlaminar separation, and residual matrix on exposed fiber surfaces. The observations at 200× and 800× were treated as qualitative fractographic evidence and were not used to establish quantitative differences in the occurrence of individual failure mechanisms among the investigated curing schedules.

A complementary semi-quantitative image analysis was performed using ImageJ software (National Institutes of Health, Bethesda, MD, USA), 1.54g version, to estimate the apparent resin coverage of exposed carbon-fiber surfaces after tensile fracture. For each selected and clearly exposed fiber, the total projected area of the visible fiber surface ($A_{total}$) was manually delineated using the polygon/freehand selection tool. The projected areas corresponding to visually identifiable residual epoxy adhering to the same fiber surface $\left(A_{resin}\right)$ were subsequently delineated and summed. The apparent resin coverage fraction $\left(A_{cov}\right)$ was calculated according to Equation (4):

(4) $$A_{cov}\left(\%\right)=\frac{A_{resin}}{A_{total}}\times 100$$

where $A_{resin}$ represents the projected area occupied by visible resin residues and $A_{total}$ represents the total projected area of the analyzed exposed fiber surface. Because the procedure is based on two-dimensional SEM projections of selected fracture-surface regions $A_{cov}$ was treated as a semi-quantitative fractographic descriptor of apparent post-fracture resin coverage and not as a direct measurement of fiber–matrix adhesion or interfacial strength.

Local apparent fiber-diameter measurements were additionally performed on clearly exposed carbon fibers in the 5000× SEM micrographs. These measurements were used only to document the characteristic dimensions of the reinforcement observed on the fracture surfaces and were not interpreted as evidence of curing-schedule-dependent changes in the intrinsic carbon-fiber diameter.

## 3. Results and Discussion

### 3.1. Tensile Tests

The tensile behavior of the carbon-fiber-reinforced laminates was evaluated using the experimental procedure described in Section 2.3.1, where five specimens were tested for each curing condition to ensure the statistical reliability and repeatability of the measured mechanical properties. The individual tensile curves are presented to illustrate the consistency of the experimental results, while the average tensile properties together with their corresponding standard deviations are summarized in Table 4.

**Figure 7:**
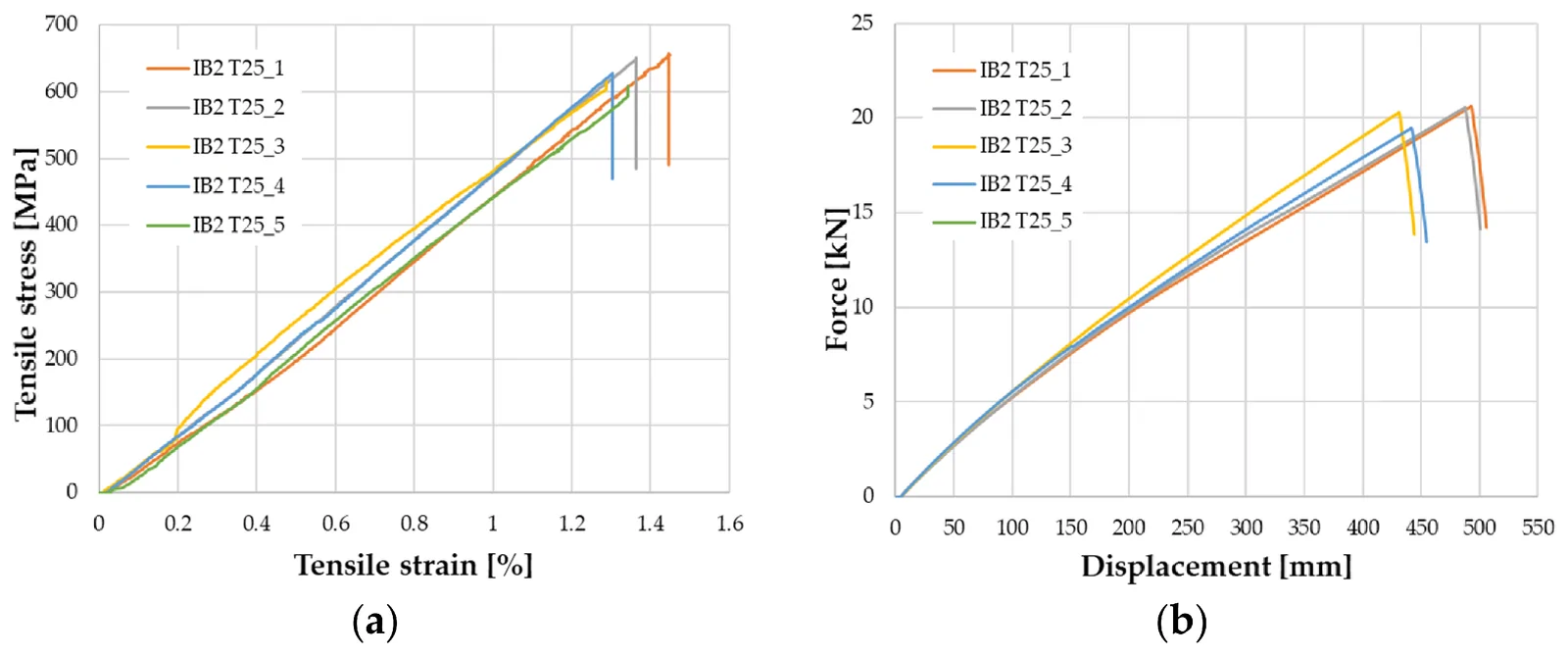
Tensile response of carbon-fiber-reinforced laminates cured at 25 °C: (**a**) tensile stress–strain and (**b**) force–displacement curves.

The tensile response of the laminates cured at 25 °C (T25) is presented in Figure 7a, while the corresponding force–displacement curves are shown in Figure 7b. The stress–strain curves exhibit an almost perfectly linear elastic response up to failure. The close overlap of the five individual curves indicates a relatively consistent tensile response among the tested specimens. Only limited variations in ultimate tensile strength and strain at maximum stress were observed, which may be associated with local variations in fiber architecture, resin distribution, and manufacturing-induced defects typical of woven CFRP laminates.

The force–displacement curves presented in Figure 7b show a similar trend, with all specimens exhibiting comparable stiffness during loading and reaching maximum loads of approximately 19–21 kN, followed by a sudden load drop associated with brittle fracture. The absence of pronounced nonlinear deformation before failure is consistent with the predominantly elastic-brittle tensile response expected for woven CFRP laminates. The characteristics of the fiber–matrix interaction and the associated failure mechanisms are further considered together with the SEM observations presented in Section 3.5. Consequently, the characteristics associated with fiber–matrix interaction and failure at their interface are not deduced exclusively from the macroscopic stress–strain response and are further evaluated through SEM fractographic observations.

The average tensile properties reported in Table 4 indicate that the laminates cured at room temperature achieved a tensile strength of 643.31 ± 26.35 MPa, a tensile modulus of 52.43 ± 14.28 GPa, a strain at maximum stress of 1.33 ± 0.08%, and an elastic strain energy density up to maximum stress of 4.27 ± 0.25 MJ/m^3^. These values provide the reference tensile response for comparison with laminates manufactured using high-temperature curing programs.

**Figure 8:**
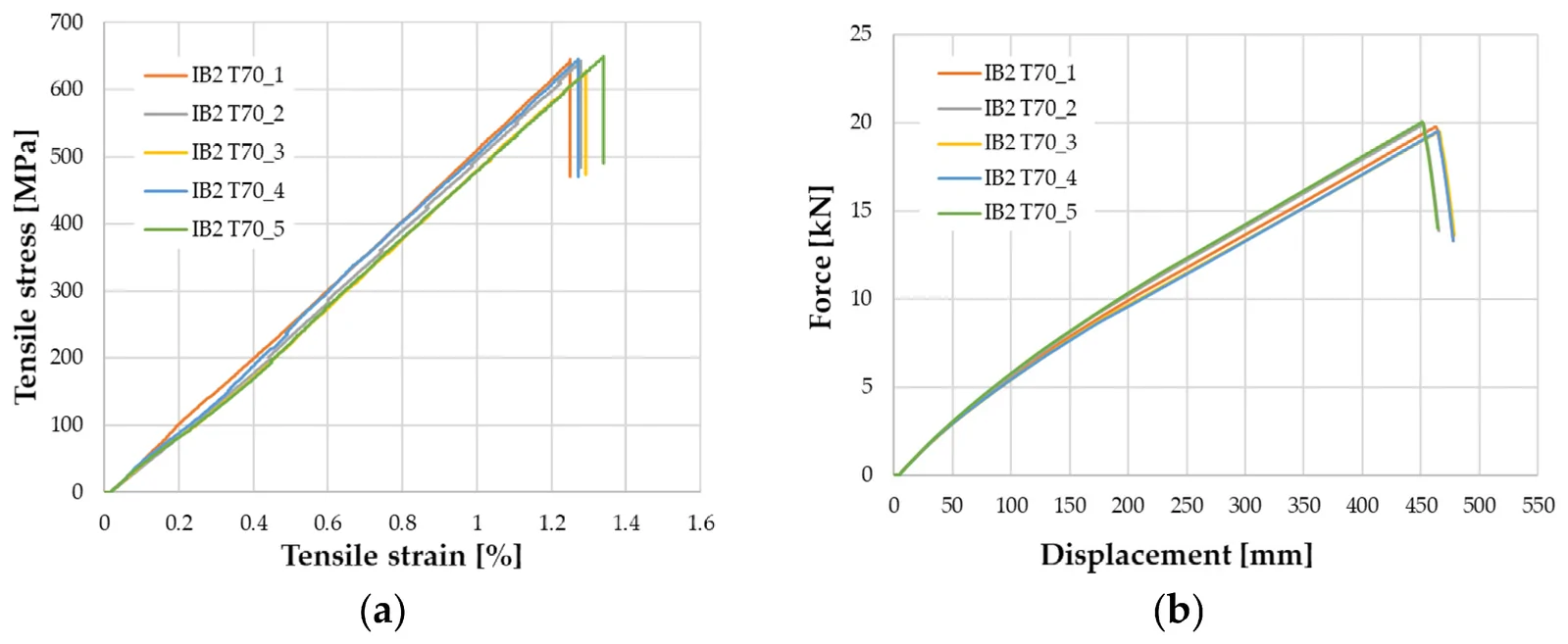
Tensile response of carbon-fiber-reinforced laminates cured at 70 °C: (**a**) tensile stress–strain and (**b**) force–displacement curves.

The tensile response of the laminates cured using a heated mould at 70 °C is presented in Figure 8a, while the corresponding force–displacement curves are illustrated in Figure 8b. Similar to the room-temperature specimens, all five specimens exhibit a predominantly linear elastic response followed by abrupt brittle fracture. The close overlap of the stress–strain curves indicates a consistent tensile response among the tested specimens, with relatively limited variability from specimen to specimen.

The force–displacement curves exhibited the same general behavior, with maximum loads close to 20 kN followed by an abrupt loss of load-bearing capacity associated with laminate fracture. Compared with T25, the T70 specimens show a slightly lower mean strain at maximum stress; however, the magnitude of this difference is small relative to the overall experimental scatter.

According to the average values summarized in Table 4, the properties of T70 laminates are lower by 1.4% in tensile strength, 12.2% in tensile modulus, 4.5% in ultimate stress–strain and 7.5% in elastic strain energy density, compared to T25 specimens. However, given the experimental scatter, especially that observed for tensile modulus, these numerical differences do not demonstrate a systematic deterioration in tensile performance for the T70 condition.

**Figure 9:**
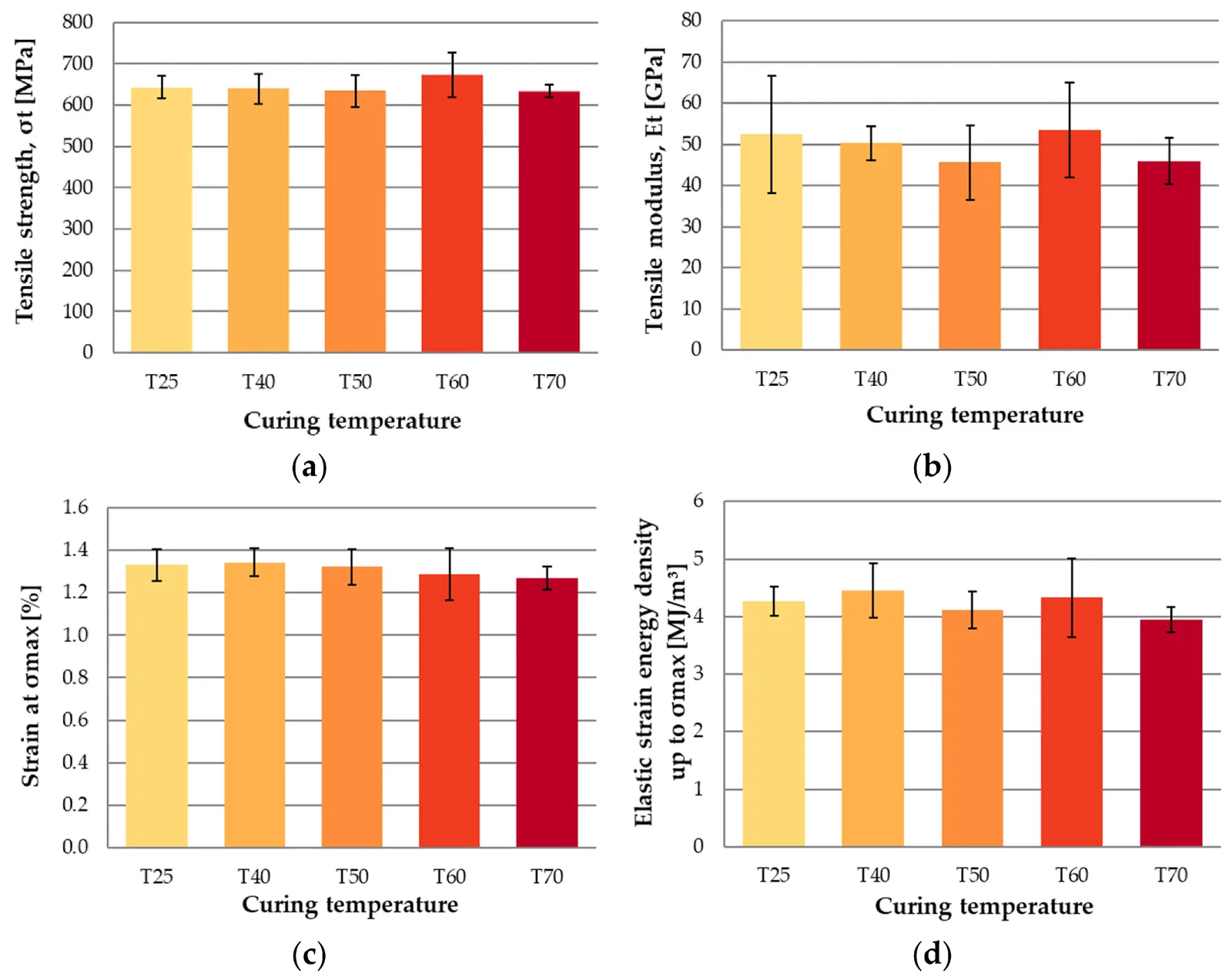
Average tensile properties obtained under the investigated curing conditions: (**a**) tensile strength, (**b**) tensile modulus, (**c**) strain at maximum stress, and (**d**) elastic strain energy density up to maximum stress.

The average tensile properties obtained under the investigated curing conditions are summarized in Table 4 and compared in Figure 9a–d. The average tensile strength remained within a relatively narrow range of approximately 634–673 MPa under all conditions (Figure 9a). T60 showed the highest average tensile strength, reaching 672.95 ± 53.60 MPa, corresponding to an increase of approximately 4.6% over T25. However, the magnitude of this difference was small compared to the experimental dispersion, as indicated by the overlapping standard deviation ranges. At T70, the average tensile strength decreased to 634.01 ± 14.68 MPa, remaining comparable to the values obtained for T25, T40 and T50.

The tensile modulus showed a similar limited dependence on the investigated curing conditions (Figure 9b). The mean values ranged from 45.58 to 53.39 GPa, with T60 having the highest numerical mean and T50 and T70 having the lowest. However, substantial overlap was observed between the standard deviation ranges, especially for T25 and T60.

The strain at maximum stress (Figure 9c) showed only minor variations between the investigated conditions, remaining within the narrow range of 1.27–1.34%. The highest average value was obtained for T40 (1.34 ± 0.07%), while the lowest was measured for T70 (1.27 ± 0.05%). Although the average values showed a slight decreasing trend between T40 and T70, the magnitude of this variation remained limited compared to the experimental dispersion. The elastic strain energy density up to maximum stress (Figure 9d) varied between 3.95 and 4.45 MJ/m^3^. The highest average value was obtained for T40 (4.45 ± 0.47 MJ/m^3^), while T70 presented the lowest value (3.95 ± 0.22 MJ/m^3^). Similar to the other tensile properties, the differences between curing conditions were relatively small compared to the experimental dispersion.

Overall, the tensile results indicate that the investigated strengthening programs produced only limited variations in the macroscopic tensile response of the woven CFRP laminates. Although T60 exhibited the highest average tensile strength and tensile modulus, these numerical maxima do not establish a distinct tensile optimum. For this reason, the tensile trends are further considered together with the HDT and DSC results, which provide complementary information on the thermal deformation under load and the calorimetric thermal response of the investigated composites. SEM observations of the fracture surface are additionally used to assess whether differences in fracture morphology and fiber–matrix interaction accompany the measured tensile response.

### 3.2. Flexural Tests

**Figure 10:**
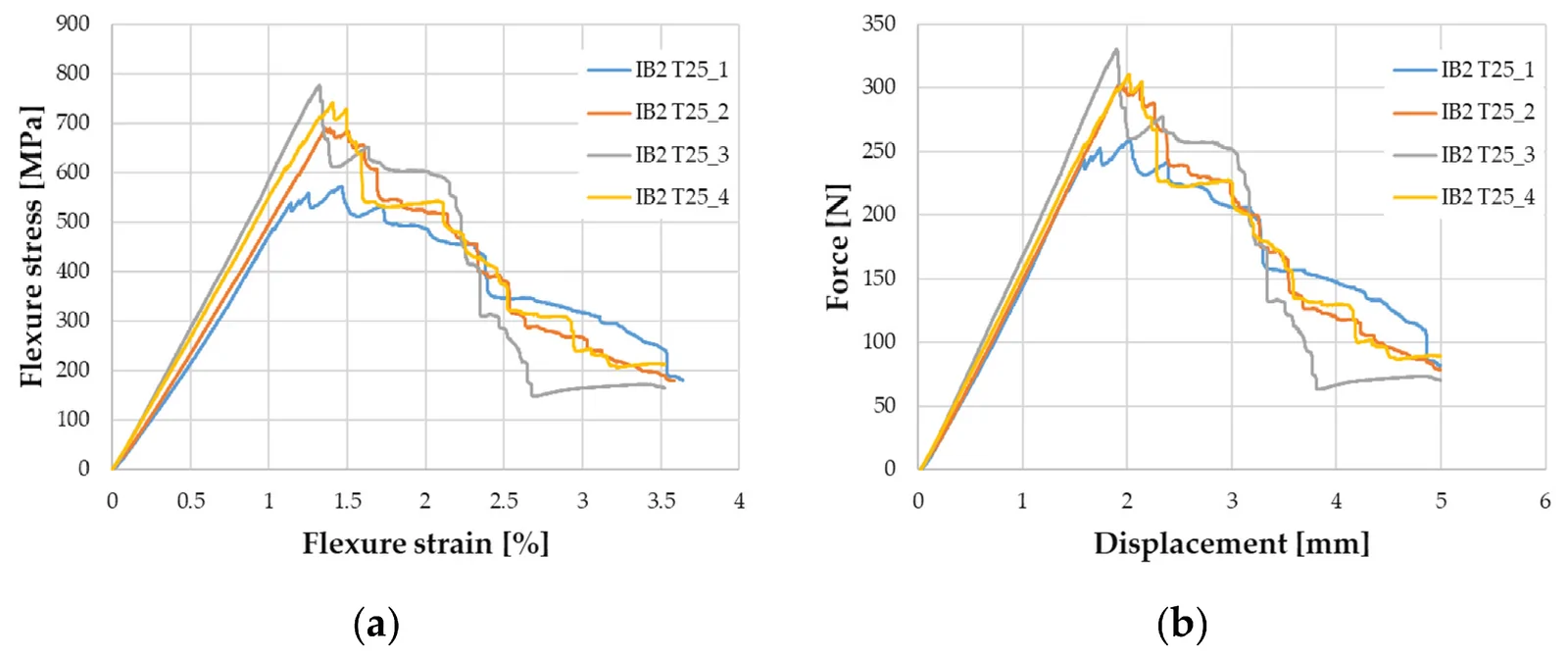
Flexural response of carbon-fiber-reinforced laminates cured at 25 °C: (**a**) flexural stress–strain curves and (**b**) force–displacement curves.

**Figure 11:**
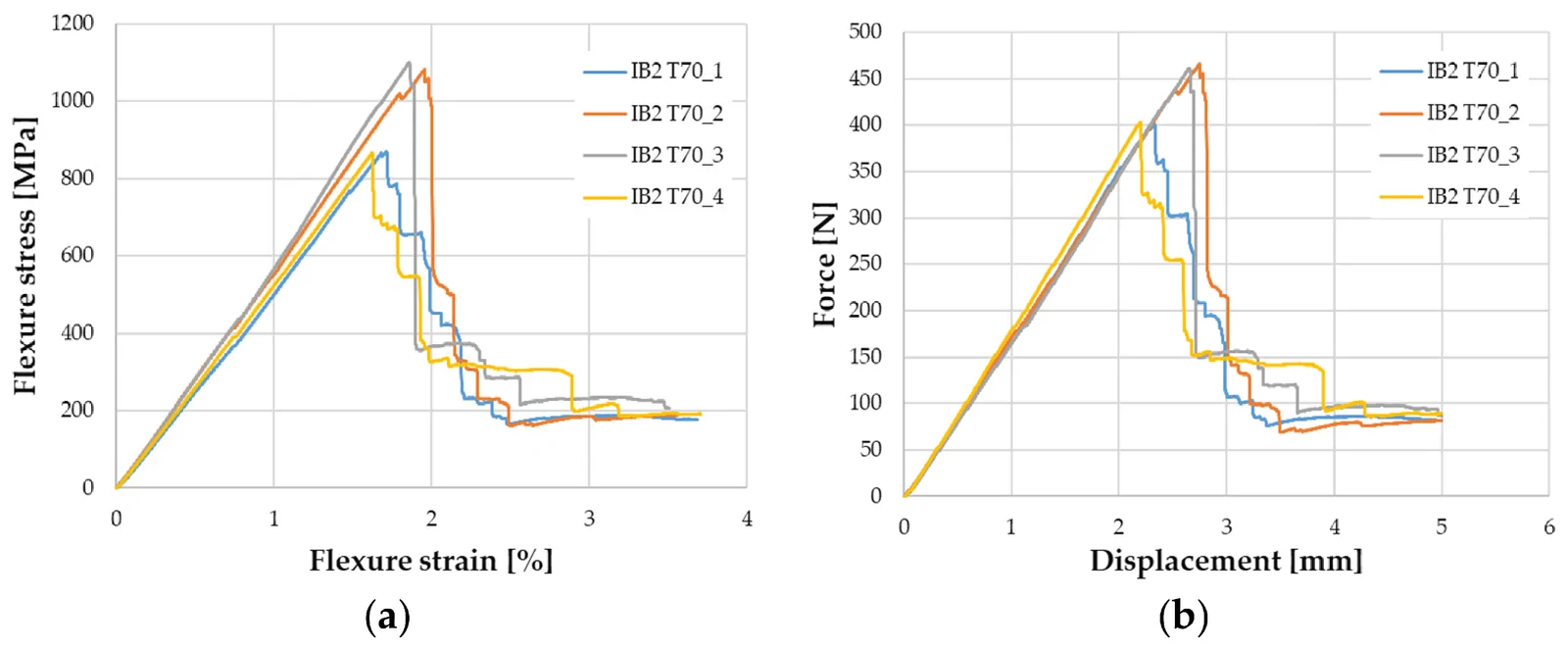
Flexural response of carbon-fiber-reinforced laminates cured at 70 °C: (**a**) flexural stress–strain and (**b**) force–displacement curves.

Five specimens were tested for each curing condition during the three-point bending experiments. For each condition, one specimen was excluded from the analysis because it deviated greatly from the mean of the others. Consequently, the bending properties reported in Table 5 and compared in Figure 10 were calculated from four valid specimens per curing condition (*n* = 4). For clarity, the four valid bending curves corresponding to the T25 and T70 conditions are shown in Figure 10 and Figure 11, respectively.

The bending response of the laminates cured at room temperature is presented in Figure 10. The stress–strain curves exhibit an approximately linear initial region corresponding to the predominantly elastic response before progressive damage develops (Figure 11a). The specimens followed similar trends in this initial region, although differences were observed in the maximum bending stress, reflecting specimen-to-specimen variability in the laminate response.

After reaching the maximum bending stress, the curves show a progressive decrease in stress, accompanied by successive decreases in load. This post-peak response indicates a progressive, rather than instantaneous, loss of load-bearing capacity and is consistent with the development of multiple damage events within the laminate. In the case of CFRP laminates, such behavior can involve matrix cracking, fiber–matrix delamination, interlaminar delamination, and progressive fiber bundle failure.

The force–displacement curves exhibit the same overall behavior, with an approximately linear increase in force followed by a gradual reduction after the maximum load was reached, as presented in Figure 10b. Although differences in the maximum load are observed between the specimens, the general shapes of the curves remain comparable, indicating generally similar macroscopic bending responses between the T25 specimens.

**Figure 12:**
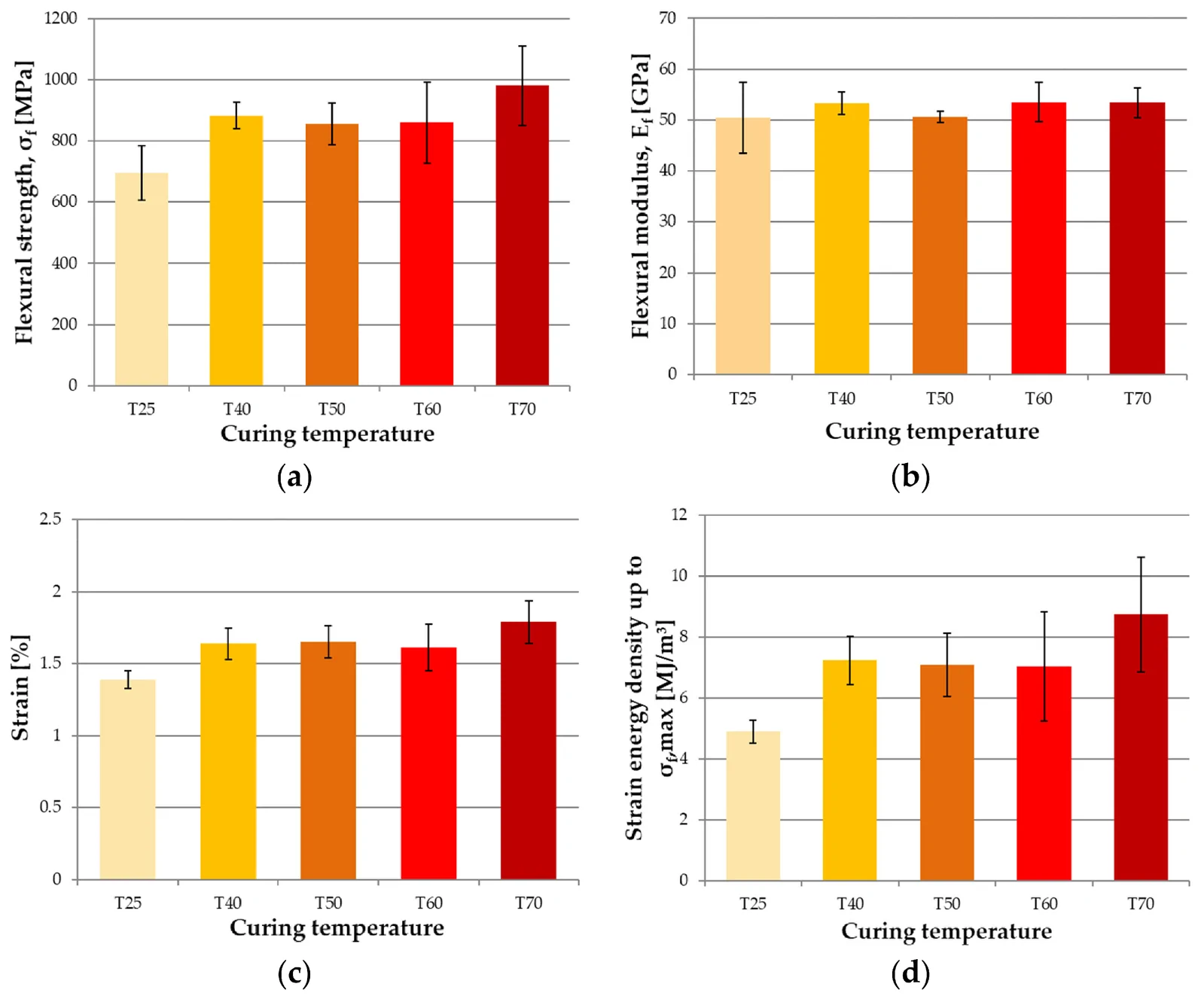
Average flexural properties obtained under the investigated curing conditions: (**a**) flexural strength, (**b**) flexural modulus, (**c**) strain at maximum flexural stress, and (**d**) strain energy density up to maximum flexural stress (mean ± standard deviation). Error bars represent standard deviations (*n* = 4).

The flexural behavior of the T70 laminates is presented in Figure 11. Similar to T25, the initial portions of the stress–strain curves are approximately linear, corresponding to a predominantly elastic response before progressive damage. T70 specimens reached higher maximum bending stresses than T25 specimens, indicating a higher load-carrying capacity under the three-point bending conditions investigated (Figure 12a).

After reaching the maximum bending stress, successive load decreases are observed, showing that the loss of load-bearing capacity occurred progressively. Compared to T25 specimens, T70 specimens exhibited both a higher maximum bending stress and a higher deformation at maximum stress, indicating that higher bending stresses were sustained at higher deformation before reaching the maximum load.

A similar tendency is observed in the force–displacement curves (Figure 11b), where the T70 specimens reached higher peak loads than the T25 specimens before entering the peak post-damage region. After the maximum load, the force decreased in successive steps, while the specimens maintained their residual load capacity, consistent with the progressive development of damage within the laminate.

The flexural strength obtained under the investigated curing conditions is shown in Figure 12a. The reference laminates cured at 25 °C showed an average flexural strength of 694.82 ± 89.00 MPa, while the T40 laminates reached 882.63 ± 43.53 MPa, corresponding to an average value approximately 27% higher. Laminates cured at 50 °C and 60 °C exhibited comparable average flexural strengths of 855.35 ± 68.13 MPa and 859.68 ± 131.76 MPa, respectively. The highest average flexural strength among the conditions investigated was obtained for T70, reaching 980.60 ± 129.03 MPa, approximately 41% higher than that of laminates cured at room temperature. In contrast to the tensile response, these results indicate a considerably stronger dependence of the flexural response on the curing schedules investigated.

The variation in the flexural modulus is shown in Figure 12b. In contrast to the flexural strength, the average flexural modulus varied only within the relatively narrow range of 50.45–53.54 GPa under the investigated conditions. The highest numerical mean was obtained for T60 (53.54 ± 3.85 GPa), while T40 and T70 showed similar values of 53.32 ± 2.19 and 53.42 ± 2.94 GPa, respectively. Given the relatively narrow range of mean values and the overlapping standard deviations, the flexural modulus appears to be considerably less sensitive to the investigated curing schedules than the flexural strength.

The maximum flexural stress–strain, shown in Figure 12c, was generally higher for the high temperature conditions than for T25. The T25 laminates reached an average value of 1.39 ± 0.06%, while T40–T60 showed values between 1.61 and 1.65%. The highest average value was measured for T70 (1.79 ± 0.15%), approximately 29% higher than for T25. Although the variation was not strictly monotonic across the investigated curing schedules, the accelerated 12 h heated-mold schedules were generally associated with higher deformation at maximum bending stress than the 24 h room-temperature reference schedule.

A similar trend was observed for the strain energy density up to the maximum bending stress (Figure 12d), which increased from 4.89 ± 0.38 MJ/m^3^ for T25 to 7.24 ± 0.80 MJ/m^3^ for T40 and remained close to 7 MJ/m^3^ for T50 and T60. The highest average value was obtained for T70 (8.75 ± 1.89 MJ/m^3^), approximately 79% higher than that of the room-temperature cured specimens. This higher average value results from the combination of the higher maximum bending stress and the corresponding higher deformation achieved by the T70 laminates.

Considering all conditions investigated, the flexural response showed more pronounced variations than the tensile response. While the axial tensile properties remained generally comparable with respect to their experimental dispersion, the high-temperature curing conditions produced higher average flexural strength, maximum bending stress–strain, and strain energy density than the T25 reference condition. This difference in sensitivity can be considered in relation to the distinct load transfer mechanisms involved in the two loading configurations. Under axial tensile loading along a principal reinforcement direction, the continuous carbon fibers provide the dominant contribution to stiffness and strength. Under three-point bending, in contrast, the laminate is simultaneously subjected to tensile and compressive stresses, along with interlaminar shear, making the macroscopic response more sensitive to the matrix, the fiber–matrix interface, and progressive damage through the laminate thickness. Consequently, the changes associated with the investigated curing schedules may be more clearly reflected in the flexural performance than in the predominantly fiber-dominated axial tensile response.

Among the curing conditions investigated, T70 exhibited the highest average flexural strength, the highest average strain at maximum bending stress, and the highest average strain energy density to maximum bending stress, while its flexural modulus remained comparable to that of the other conditions. Therefore, T70 can be identified as the condition exhibiting the highest average values for several flexural performance metrics within the investigated temperature–time schedules. However, the current results do not establish 70 °C as the overall optimum curing temperature. The relationship between these mechanical trends and the thermal response of the laminates is further considered in conjunction with the HDT and DSC results.

### 3.3. HDT Tests

**Figure 13:**
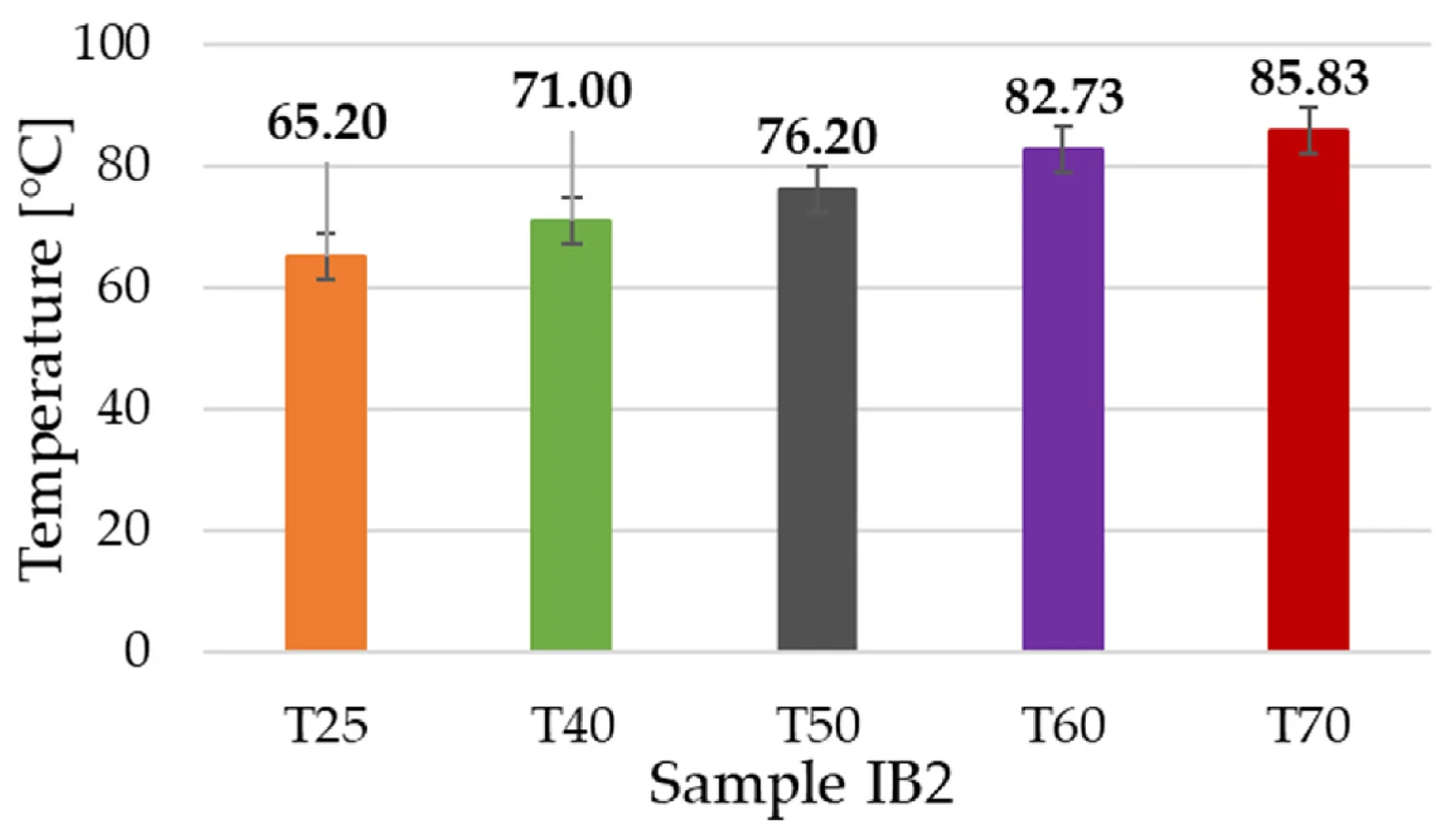
HDT of the bio-based CFRP laminates obtained under the investigated curing schedules.

Heat deflection temperature (HDT) is an engineering parameter that characterizes the temperature at which a material reaches a specified deformation under a prescribed mechanical load. Therefore, for CFRP laminates, HDT provides a comparative measure of the resistance to thermally induced deformation under specific test loading conditions. HDT increased progressively among the investigated curing conditions, as shown in Figure 13. T25 showed the lowest average HDT of 65.20 °C, while average values of 71.00, 76.20, 82.73, and 85.83 °C were obtained for T40, T50, T60, and T70, respectively. Thus, T70 showed the highest average HDT among the investigated conditions, approximately 31.6% higher than T25. This progressive increase indicates that the laminates produced using the high-temperature curing programs reached the prescribed deformation criterion at progressively higher temperatures. It should be emphasized that HDT is a single-point, load-dependent engineering measurement and does not provide a direct measure of polymer-network rigidity or crosslink density. Therefore, the observed differences in HDT among the investigated curing schedules cannot by themselves distinguish among possible contributions from changes in crosslinking, free volume, network topology, residual stresses, or other processing-induced effects. In the present study, the HDT results are consequently interpreted only as evidence of differences in resistance to deformation under the prescribed loading and heating conditions.

Although HDT increased over the entire range investigated, the increase between T60 and T70 was smaller (+3.10 °C) than that observed between T50 and T60 (+6.53 °C). This reduction in incremental increase may indicate a decreased sensitivity of the HDT response between the T60 and T70 curing schedules.

The relatively limited experimental scatter indicates good repeatability of the HDT measurements within each curing condition. The laminates cured at room temperature exhibited slightly greater variability than the specimens produced using the heated mold curing programs. Overall, the HDT results show that the laminates reached the prescribed deformation criterion at progressively higher temperatures under the specific loading and heating conditions of the HDT test.

### 3.4. DSC Analysis

**Figure 14:**
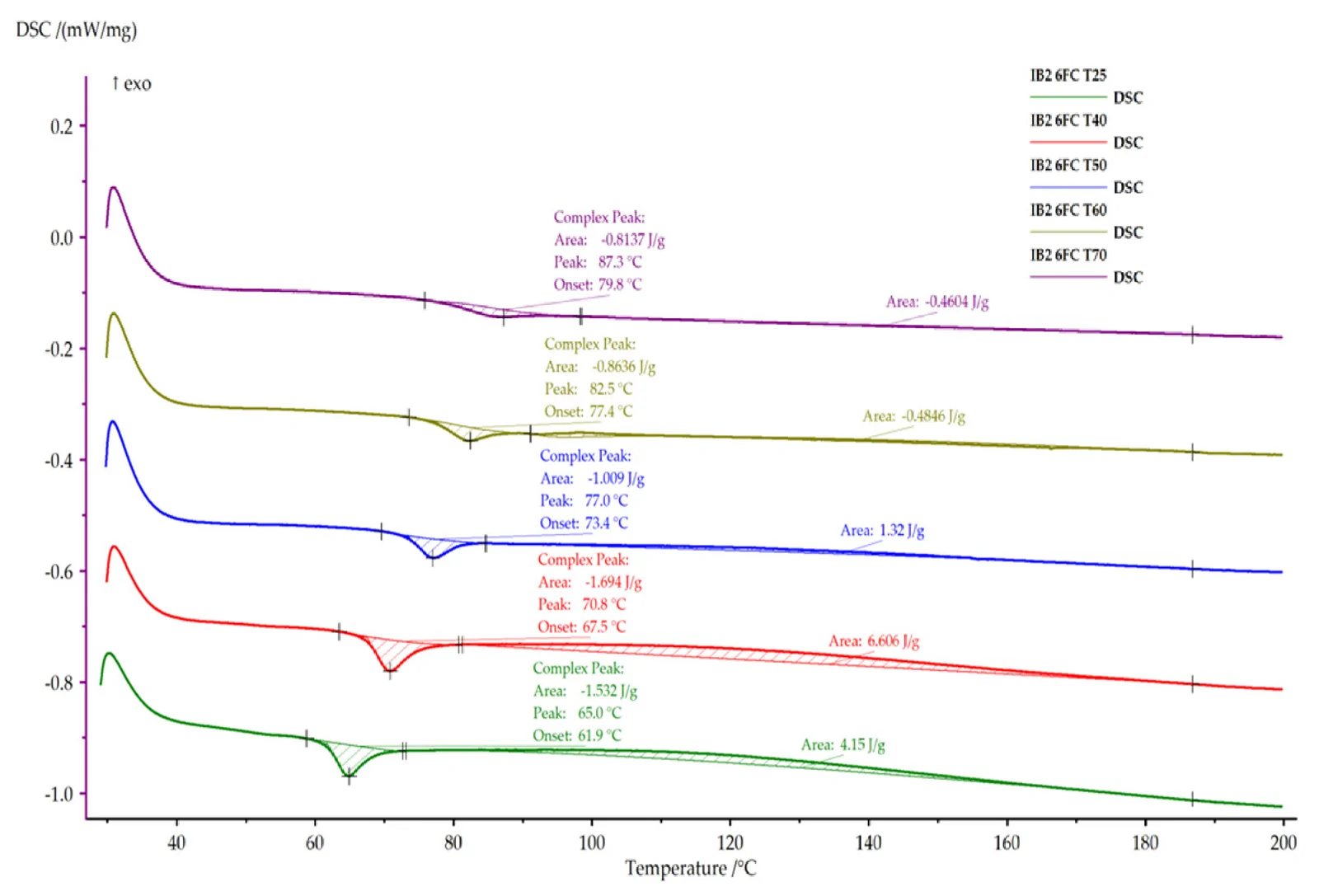
First-heating DSC curves of the bio-based CFRP laminates obtained under the investigated curing schedules, showing the complex calorimetric response associated with the as-cured thermal history.

DSC measurements were performed on approximately 8 mg specimens extracted from the cured CFRP laminates. During the first heating cycle, a complex thermal event was observed between approximately 60 and 90 °C for all conditions investigated, as shown in Figure 14. The peak temperature changed from 65.0 °C for T25 to 70.8, 77.0, 82.5, and 87.3 °C for T40, T50, T60, and T70, respectively. Similarly, the onset temperature changed from 61.9 °C for T25 to 79.8 °C for T70. These results demonstrate a clear dependence of the calorimetric response on the first heating on the curing schedules investigated.

Since the first heating cycle was performed on the laminates in their cured state, the calorimetric response reflects the thermal and processing history established prior to DSC testing. The progressive shift in the observed thermal characteristic towards higher temperatures therefore indicates differences between the curing programs investigated.

The morphology suggests the presence of overlapping calorimetric contributions associated with the curing state of the epoxy matrix. These may include the glass transition region, enthalpic relaxation and residual curing reactions occurring during heating. Because these contributions overlap, the integrated area of this event was not used to calculate a quantitative degree of curing.

The absolute magnitude of the integrated area associated with the complex thermal event was approximately 1.53 J/g for T25, 1.69 J/g for T40, 1.01 J/g for T50, 0.86 J/g for T60 and 0.81 J/g for T70. Although a general reduction in magnitude was observed from T40 to T70, the variation was not strictly monotonic across all curing conditions, as T40 exhibited a slightly higher absolute value than T25. Furthermore, the DSC specimens consisted of small fragments extracted from CFRP laminates, and local variations in the fiber-to-resin ratio may affect the apparent heat flux signal when normalized to the total mass of the specimen. For these reasons, the integrated areas are interpreted qualitatively and are not treated as direct measurements of residual reaction enthalpy or degree of curing.

The large additional calorimetric contribution was more pronounced for T25–T50 and became considerably less evident for T60 and T70. This observation further indicates that the thermal response after curing differed between the investigated processing schemes.

Overall, the first heating DSC curves demonstrate that the investigated curing schemes produced different calorimetric responses after curing. The thermal characteristic shifted towards higher temperatures, while the large additional contribution became less pronounced for the higher temperature curing schemes. Therefore, these observations are interpreted qualitatively and are not considered a quantitative determination of the degree of curing.

**Figure 15:**
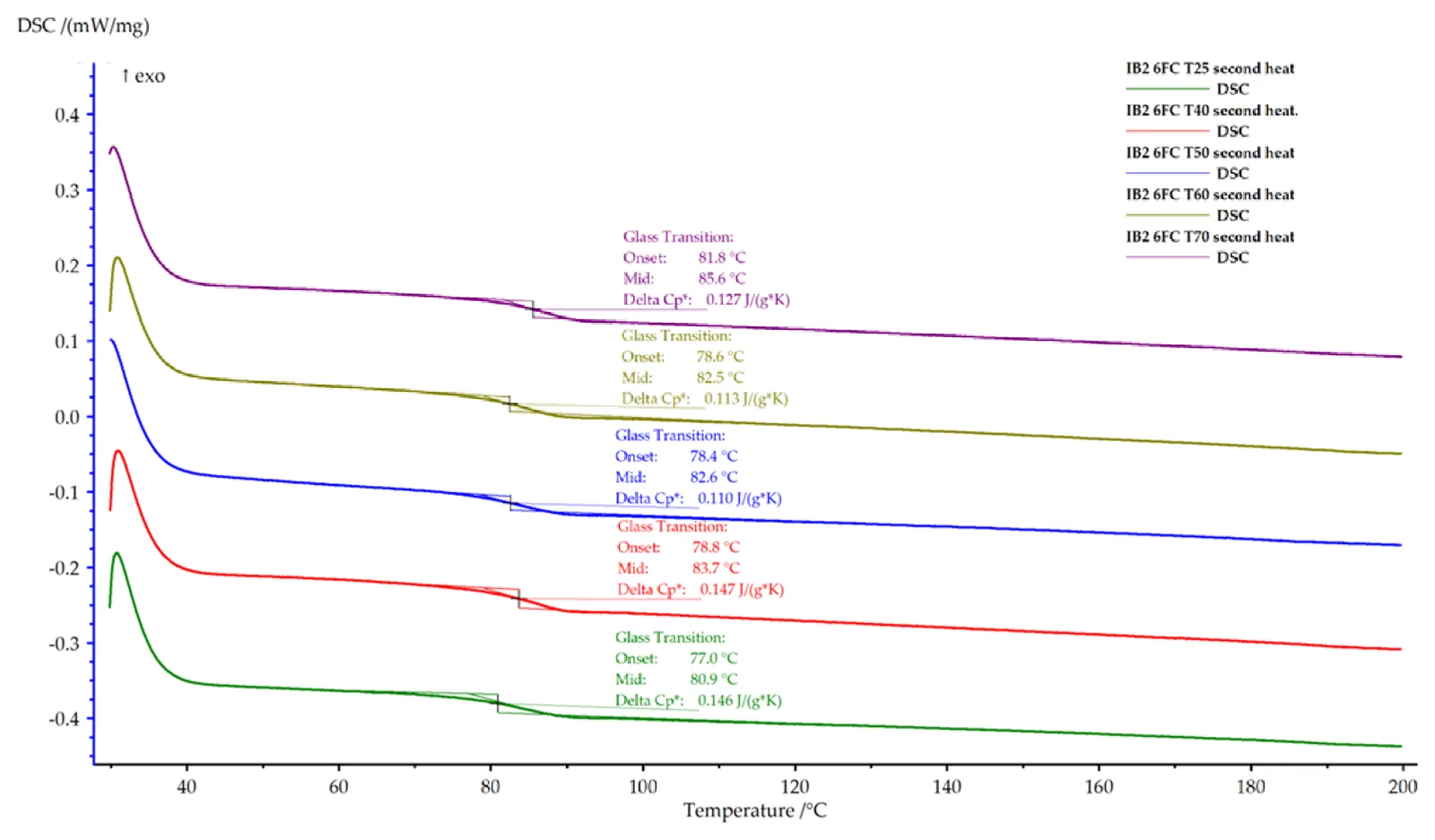
Second-heating DSC curves of the bio-based CFRP laminates showing the determination of the glass transition temperature (Tg) after removal of the previous thermal history.

The second heating curves exhibited a substantially simpler calorimetric response, characterized by a distinct glass transition stage and the absence of the pronounced complex features observed during the first heating cycle, as shown in Figure 15. During the first DSC cycle, all specimens were subjected to the same thermal excursion up to 200 °C. This common thermal treatment greatly reduced the differences associated with the initial thermal history and may also have allowed residual curing reactions to occur before the second heating cycle. Therefore, the glass transition temperature was determined from the midpoint of the heat capacity stage during the second heating cycle.

The Tg values measured at the midpoint were 80.9 °C for T25, 83.7 °C for T40, 82.6 °C for T50, 82.5 °C for T60 and 85.6 °C for T70. Therefore, the values occupied a relatively narrow range of 80.9–85.6 °C and did not vary monotonically across the initial curing schedules (Figure 15). T70 showed the highest Tg value measured at the midpoint, while T40, T50 and T60 differed by only about 1.2 °C.

The measured heat capacity increments (ΔCp) ranged from 0.110 to 0.147 J/(g K). Since these values were calculated using the total mass of the composite specimens and not just the mass of the epoxy matrix, they only provide a qualitative basis for comparison between the laminates investigated. The carbon fiber fraction reduces the apparent magnitude of the heat capacity change, and therefore, direct comparison with ΔCp values reported for pure epoxy systems should be avoided. Overall, the DSC results show that the investigated curing programs influenced the post-cure calorimetric response observed during the first heating cycle, while the Tg values from the second heating converged within a relatively narrow range of 80.9–85.6 °C after a common DSC thermal cycle. Therefore, the DSC measurements provide information on the calorimetric response and glass transition of the epoxy matrix in the composite laminates, but do not constitute a quantitative analysis of curing kinetics or degree of cure. Importantly, neither the DSC response nor the measured Tg values are interpreted as evidence of thermal degradation resistance. DSC and HDT provide distinct calorimetric and engineering deformation information, respectively, and their combined use should not be interpreted as a substitute for direct characterization of thermal degradation behavior or temperature-dependent viscoelastic properties.

### 3.5. Results of SEM Fractographic Analysis

**Figure 16:**
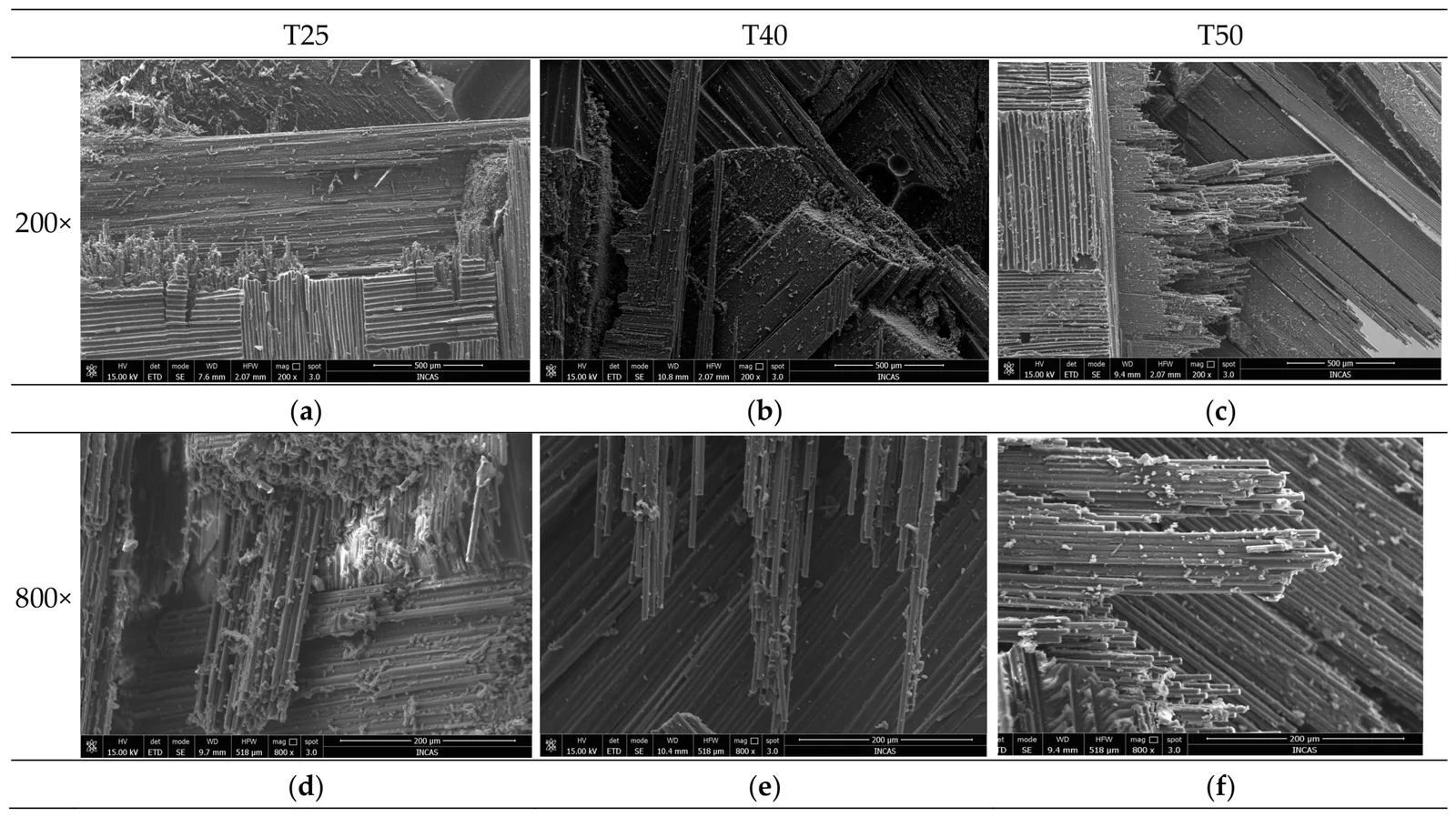
SEM micrographs of tensile-fracture surfaces of CFRP laminates obtained under the investigated curing conditions: (**a**,**d**) T25; (**b**,**e**) T40; (**c**,**f**) T50. Images in the upper and lower rows were acquired at nominal magnifications of 200× and 800×, respectively.

**Figure 17:**
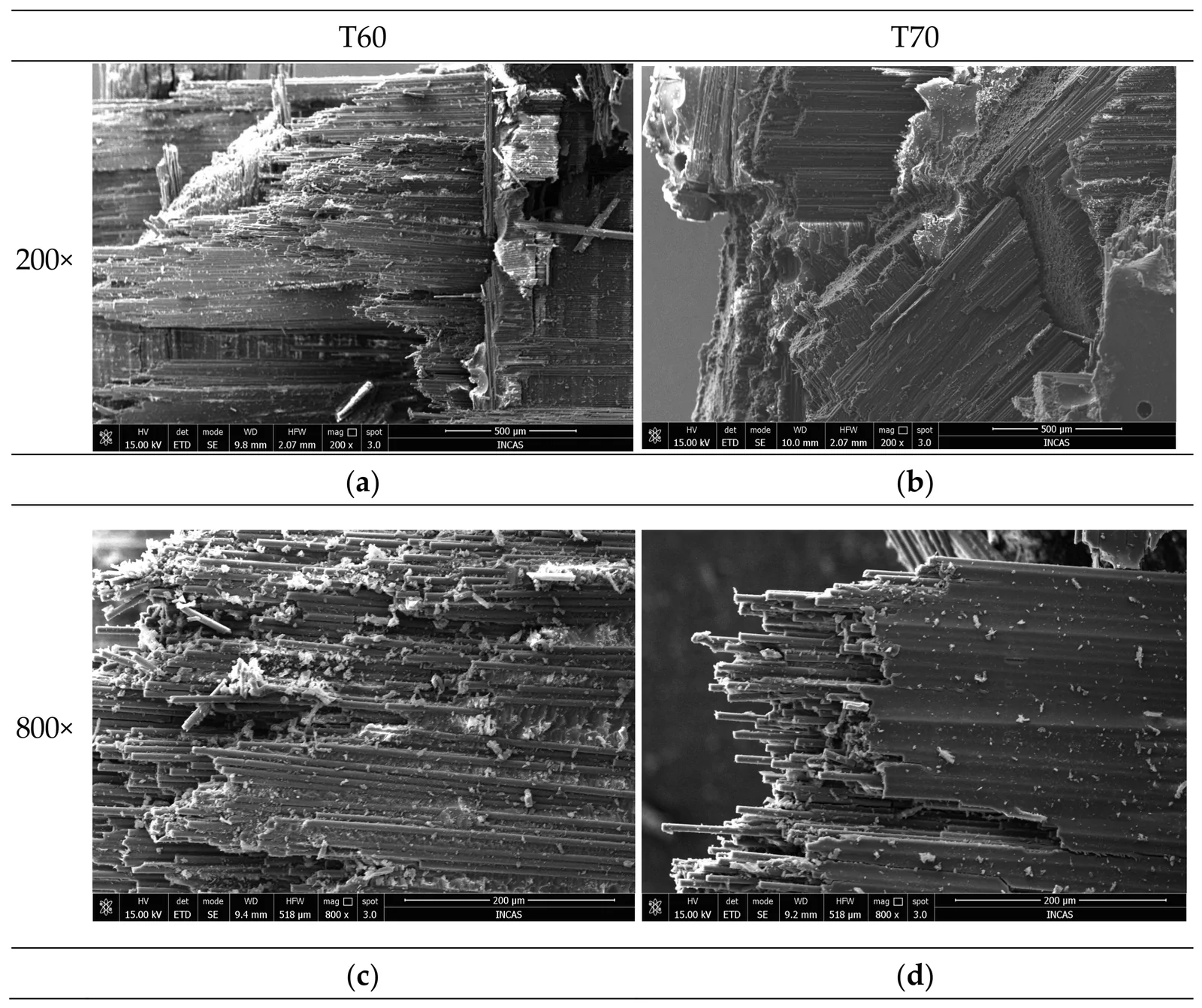
SEM micrographs of tensile-fracture surfaces of CFRP laminates obtained under the investigated curing conditions: (**a**,**c**) T60; (**b**,**d**) T70. Images in the upper and lower rows were acquired at nominal magnifications of 200× and 800×, respectively.

SEM fractography was performed on tensile-fracture surfaces from all five curing conditions in order to compare the dominant failure features and their possible relationship with the measured tensile response. Representative micrographs acquired at 200× and 800× are presented in Figure 16 and Figure 17.

Across all curing conditions, the fracture surfaces exhibited a mixed failure morphology involving fiber-bundle fracture, local fiber pull-out, matrix cracking, fiber–matrix separation, and interlaminar damage. The coexistence of these mechanisms is consistent with the complex fracture behavior expected for multidirectional woven CFRP laminates. The T25 specimens exhibited fractured fiber bundles, exposed fibers, and local interlaminar separation. At T40, similar mechanisms remained evident, together with localized cavities and elongated exposed fibers consistent with local fiber pull-out and interfacial separation. The T50 fracture surfaces showed more pronounced longitudinal splitting along the fiber-bundle direction, accompanied by extensive fiber exposure and local delamination.

T60 also exhibited the highest numerical mean values of tensile strength and tensile modulus among the investigated conditions. Nevertheless, because the differences in tensile properties were small relative to the experimental scatter, the observed fracture morphology is not interpreted as a mechanistic explanation for the numerical tensile maximum at T60. Instead, the SEM observations provide complementary qualitative information on the post-fracture morphology, while the semi-quantitative resin-coverage analysis presented below provides an additional descriptor of residual matrix on exposed fiber surfaces.

**Figure 18:**
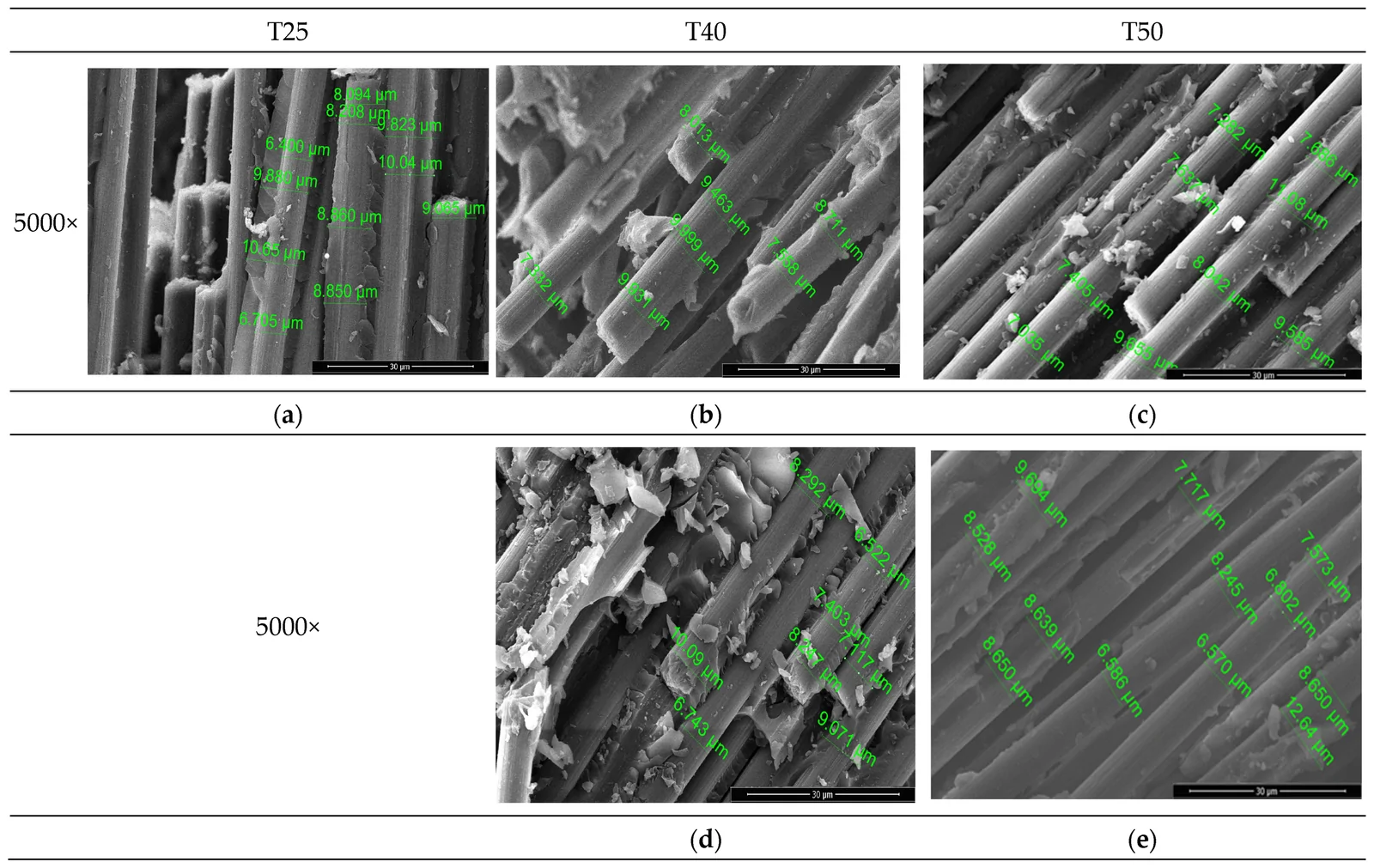
Representative SEM micrographs at 5000× magnification showing local apparent carbon-fiber diameter measurements for CFRP laminates obtained under the investigated curing schedules: (**a**) T25, (**b**) T40, (**c**) T50, (**d**) T60, and (**e**) T70. The markers indicate individual local diameter measurements performed on exposed carbon fibers. Scale bar: 30 µm.

Local measurements performed on the 5000× SEM micrographs showed that the apparent carbon-fiber diameters remained within a comparable micrometric range across the examined fracture surfaces (Figure 18). Because the same carbon-fiber reinforcement was used for all laminates, these measurements were performed only to document the characteristic dimensions of the fibers examined at high magnification and were not interpreted as a curing-schedule-dependent response.

To complement the qualitative fractographic observations, a semi-quantitative ImageJ analysis was performed to estimate the apparent resin coverage fraction ${(A}_{cov})$ on exposed carbon-fiber surfaces. As summarized in Table 6, the measured $\left(A_{cov}\right)$ values were 45.0% for T25, 51.3% for T40, 51.6% for T50, 56.8% for T60, and 55.6% for T70. Within the analyzed SEM regions, the heated-mold conditions therefore exhibited greater apparent residual resin coverage on the examined exposed fiber surfaces than the T25 reference, with the highest numerical value observed for T60 and a similar value for T70.

The $A_{cov}$ results complement the qualitative observation of residual matrix on exposed fibers and fiber bundles. However, this analysis does not quantify the relative occurrence of fiber pull-out, fiber fracture, delamination, or other individual fracture mechanisms. Furthermore, because $A_{cov}$ is derived from two-dimensional SEM images of selected fracture-surface regions, it should not be interpreted as a direct measurement of fiber–matrix adhesion or interfacial strength. Accordingly, the SEM observations are used to characterize post-fracture morphology rather than to establish definitive mechanisms responsible for the differences in macroscopic mechanical properties among the investigated curing schedules.

### 3.6. Limitations

Several limitations of the present study should be acknowledged. First, the fiber volume fraction was estimated using a geometric–gravimetric approach rather than measured directly, and the void content was not independently quantified. Consequently, possible local variations in porosity or resin distribution cannot be fully separated from the effects of the investigated curing conditions. Second, dynamic mechanical analysis (DMA) was not performed; therefore, storage modulus, loss modulus, damping behavior, and the temperature-dependent viscoelastic response of the laminates were not evaluated. Accordingly, the HDT and DSC results are not interpreted as measures of dynamic mechanical behavior or polymer-network rigidity, intrinsic thermal stability, or thermal degradation resistance. Third, curing temperature and curing duration were not varied independently: T25 represents the manufacturer-recommended 24 h room-temperature reference, whereas T40–T70 represent accelerated 12 h heated-mold schedules. Consequently, differences between T25 and the heated-mold conditions cannot be attributed specifically to curing temperature, but must be interpreted as effects of the combined curing schedules. Fourth, no conventional petroleum-based epoxy control was experimentally investigated in the present study. Therefore, the measured mechanical and thermal-deformation properties of the IB2-based CFRP laminates should not be interpreted as demonstrating equivalent or superior performance relative to conventional petroleum-based epoxy composites. Such a comparison would require a matched reference laminate manufactured using the same carbon-fiber architecture, fiber volume fraction, manufacturing route, curing conditions, specimen preparation, and testing procedures. Accordingly, any manufacturer-reported or literature data concerning conventional epoxy systems are considered contextual information only and not experimental evidence of comparative performance in the present study. Finally, the SEM-based resin-coverage analysis is inherently local and two-dimensional. The calculated $A_{cov}$ values characterize selected exposed fiber surfaces within the examined tensile-fracture regions and do not constitute a comprehensive quantitative analysis of the relative occurrence of fiber fracture, fiber pull-out, delamination, or other failure mechanisms throughout the laminate. Therefore, these values are treated as semi-quantitative fractographic descriptors rather than bulk measures of fiber–matrix interfacial properties. Future work should include direct void-content measurements, controlled curing schedules with identical durations, cure-kinetics analysis, and DMA to further clarify the relationships between processing, matrix state, and laminate performance and a matched conventional petroleum-based epoxy reference manufactured with the same carbon-fiber architecture, fiber volume fraction, manufacturing route, and testing conditions, together with controlled curing schemes.

## 4. Conclusions

This study evaluated a practical out-of-autoclave processing strategy for CFRP laminates fabricated using the commercially available biomass-based infusible epoxy resin IB2. The manufacturer’s recommended room-temperature curing program (T25, 24 h) was used as a reference and compared to accelerated mold heating programs at 40–70 °C with a reduced curing time of 12 h. The main findings are:

- Accelerated heated mold curing programs maintained an axial tensile response generally comparable to the manufacturer’s recommended room-temperature reference. The average tensile strength ranged from 634.01 to 672.95 MPa across all conditions investigated. T60 exhibited the highest number average tensile strength (672.95 ± 53.60 MPa) and tensile modulus (53.39 ± 11.53 GPa); however, these differences were small relative to the experimental spread and therefore do not establish a distinct tensile optimum at 60 °C. Overall, the tensile response was relatively insensitive to the curing programs investigated.
- The flexural response was considerably more sensitive to the curing programs investigated. The average flexural strength increased from 694.82 ± 89.00 MPa for T25 to 980.60 ± 129.03 MPa for T70, corresponding to an average value approximately 41% higher, while the flexural modulus remained in the relatively narrow range of 50.45–53.54 GPa. T70 also exhibited the highest average strain at maximum bending stress and the highest strain energy density up to maximum bending stress. The greater sensitivity of the flexural response may be related to the greater influence of matrix-, interface-, and interlaminar-related deformation and damage mechanisms under three-point bending compared with the predominantly fiber-dominated axial tensile response. These results identify T70 as the highest-performing condition for several flexural metrics within the investigated temperature–time schedules, but do not establish 70 °C as an optimum curing temperature.
- SEM fractography revealed variations in the post-fracture morphology among the investigated curing schedules. Complementary semi-quantitative ImageJ analysis identified differences in the apparent resin coverage of exposed carbon-fiber surfaces, providing an additional descriptor of the observed fracture-surface characteristics without implying a direct measure of fiber–matrix interfacial strength.
- The HDT values differed systematically among the investigated curing schedules, increasing from 65.20 °C for T25 to 85.83 °C for T70, corresponding to an increase of approximately 31.6%. Complementary DSC measurements revealed a pronounced dependence of the first-heating calorimetric response on the initial curing schedule, while the midpoint Tg values determined during the second heating occupied a relatively narrow range of 80.9–85.6 °C after all specimens had undergone the same DSC thermal cycle. These HDT and DSC results provide distinct information and are not interpreted as evidence of dynamic mechanical behavior, intrinsic thermal stability, polymer-network rigidity, or thermal degradation resistance. Furthermore, the DSC results do not establish the degree of cure or demonstrate complete cure for any of the investigated curing schedules.
- The results demonstrate the feasibility of reducing the cure time from 24 h to 12 h using heated mold processing while maintaining generally comparable tensile properties and achieving higher average flexural performance and increased resistance to thermal deformation under the prescribed HDT test conditions for the elevated-temperature curing schedules. This reduction in cure time is relevant to off-autoclave manufacturing, where shorter mold occupancy times can contribute to increased production yield.
- The present results are limited to comparisons among curing schedules for the IB2-based CFRP system investigated. Because no matched petroleum-based epoxy control was included, the results do not establish comparable, superior, or inferior performance of IB2-based laminates relative to conventional petroleum-based epoxy composites. A direct assessment would require reference laminates produced with the same carbon-fiber architecture, fiber volume fraction, manufacturing route, and testing conditions.
- The results support the further development of accelerated processing routes for biomaterial-based CFRP laminates for lightweight structural applications. Further investigations should include controlled curing schemes with identical durations, direct void content measurements, curing kinetics and dynamic mechanical analyses, as well as fatigue, impact and damage tolerance testing, environmental conditioning, interlaminar characterization, long-term thermal exposure, and component-level validation prior to structural aerospace application.

## Figures and Tables

**Table 1:** Main physical and processing properties of the raw materials used in this study.

Material	Property	Value
IB2 Bio Epoxy Resin	Plant-derived content	~38%
	Mixed viscosity (20 °C)	290 mPa·s
	Mixed density (20 °C)	1.12 g/cm^3^
	Mixing ratio	100:22 (wt.)
Carbon fabric	Weave	2 × 2 twill
	Areal weight	200 g/m^2^
	Fiber density	1.80 g/cm^3^
	Number of plies	6

**Table 2:** Average dimensions and mass of the HDT specimens and estimated fiber volume fraction.

Sample	Length [mm]	Width [mm]	Thickness [mm]	Mass [g]	Fiber Mass, $m_{f}$ [g]	Resin Mass, $m_{r}$ [g]	Fiber Volume Fraction, $V_{f}$ [%]
T25 (25 °C)	100.24	31.98	1.23	5.98	3.847	2.133	52.9
T40 (40 °C)	100.30	32.00	1.25	5.89	3.851	2.039	54.0
T50 (50 °C)	100.20	31.99	1.24	5.99	3.846	2.144	52.7
T60 (60 °C)	100.15	31.99	1.25	5.86	3.845	2.015	54.3
T70 (70 °C)	100.03	31.90	1.23	5.78	3.829	1.951	55.0

**Table 3:** Characterization techniques, experimental equipment and corresponding testing standards used in this study.

Characterization Method	Equipment	Standard
Tensile testing	Instron 5982 + Video Extensometer	ISO 527-4 [24]
Flexural testing	Instron 5982	ISO 14125 [25]
HDT testing	Qualitest HDT/Vicat	ASTM D648 [26]
Differential scanning calorimetry (DSC)	NETZSCH DSC 300 Caliris^®^ Classic	ISO 11357 [27,28]
SEM observations	Quanta 250 SEM	-

**Table 4:** Average tensile properties of laminates obtained under the investigated curing schedules, reported as mean ± standard deviation.

Curing Schedule	Tensile Strength, σt [MPa]	Tensile Modulus, Et [GPa]	Strain at σmax [%]	Elastic Strain Energy Density Up to σmax [MJ/m^3^]
T25	643.31 ± 26.35	52.43 ± 14.28	1.33 ± 0.08	4.27 ± 0.25
T40	639.78 ± 36.07	50.28 ± 4.07	1.34 ± 0.07	4.45 ± 0.47
T50	634.58 ± 38.63	45.58 ± 9.07	1.32 ± 0.08	4.12 ± 0.33
T60	672.95 ± 53.60	53.39 ± 11.53	1.28 ± 0.12	4.33 ± 0.68
T70	634.01 ± 14.68	46.01 ± 5.63	1.27 ± 0.05	3.95 ± 0.22

**Table 5:** Average flexural properties of CF/IB2 laminates obtained under the investigated curing conditions, reported as mean ± standard deviation (*n* = 4).

Curing Schedule	Flexural Strength, σ_f_ [MPa]	Flexural Modulus, E_f_ [GPa]	Strain at σ_f_, [%]	Strain Energy Density Up to σ_f_ [MJ/m^3^]
T25	694.82 ± 89.00	50.45 ± 6.95	1.39 ± 0.06	4.89 ± 0.38
T40	882.63 ± 43.53	53.32 ± 2.19	1.64 ± 0.11	7.24 ± 0.80
T50	855.35 ± 68.13	50.59 ± 1.06	1.65 ± 0.11	7.09 ± 1.04
T60	859.68 ± 131.76	53.54 ± 3.85	1.61 ± 0.16	7.04 ± 1.80
T70	980.60 ± 129.03	53.42 ± 2.94	1.79 ± 0.15	8.75 ± 1.89

**Table 6:** SEM-based apparent resin coverage fraction ($A_{cov}$) on exposed carbon-fiber surfaces after tensile fracture for the investigated curing schedules.

Curing Schedule	T25	T40	T50	T60	T70
Apparent resin coverage fraction, $A_{cov}$ [%]	45	51.3	51.6	56.8	55.6

## Data Availability

The original contributions presented in the study are included in the article; further inquiries can be directed to the corresponding author or to the following e-mail address larisa_anda.stroe@upb.ro.

## Abbreviations

The following abbreviations are used in this manuscript:

CFRP	Carbon-Fiber-Reinforced Polymer
DSC	Differential Scanning Calorimetry
HDT	Heat Deflection Temperature
SEM	Scanning Electron Microscopy
UAV	Unmanned Aerial Vehicles
